# Metabolite and Transcriptome Profiling on Xanthine Alkaloids-Fed Tea Plant (*Camellia sinensis*) Shoot Tips and Roots Reveal the Complex Metabolic Network for Caffeine Biosynthesis and Degradation

**DOI:** 10.3389/fpls.2020.551288

**Published:** 2020-09-09

**Authors:** Cheng Deng, Xiuping Ku, Lin-Lin Cheng, Si-an Pan, Limao Fan, Wei-Wei Deng, Jian Zhao, Zheng-Zhu Zhang

**Affiliations:** State Key Laboratory of Tea Plant Biology and Utilization, Anhui Agricultural University, Hefei, China

**Keywords:** purine alkaloids, caffeine, biosynthesis, metabolic pathway, feeding, degradation

## Abstract

While caffeine is one of the most important bioactive metabolites for tea as the most consumed non-alcohol beverage, its biosynthesis and catabolism in tea plants are still not fully understood. Here, we integrated purine alkaloid profiling and transcriptome analysis on shoot tips and roots fed with caffeine, theophylline, or theobromine to gain further understanding of caffeine biosynthesis and degradation. Shoot tips and roots easily took up and accumulated high concentrations of alkaloids, but roots showed much faster caffeine and theophylline degradation rates than shoot tips, which only degraded theophylline significantly but almost did not degrade caffeine. Clearly feedback inhibition on caffeine synthesis or inter-conversion between caffeine, theophylline, and theobromine, and 3-methylxanthine had been observed in alkaloids-fed shoot tips and roots, and these were also evidenced by significant repression of *TCS* and *MXMT* genes critical for caffeine biosynthesis. Among these responsively repressed genes, two highly expressed genes *TCS-4* and *TCS-8* were characterized for their enzyme activity. While we failed to detect TCS-4 activity, TCS-8 displayed *N*-methyltransferase activities towards multiple substrates, supporting the complex metabolic network in caffeine biosynthesis in tea plants since at least 13 TCS-like *N*-methyltransferase genes may function redundantly. This study provides new insight into complex metabolic networks of purine alkaloids in tea plants.

## Highlights

Tea plant roots have stronger degradation activity on both caffeine and theophylline. Caffeine feeding to tea plant shoot tips and roots resulted in feedback inhibitions in the biosynthetic gene expression and accumulation of theobromine, whereas theophylline feeding resulted in both caffeine biosynthesis and theophylline degradation through 3-methylxanthine. Both metabolite profiling, transcriptome analysis, and enzyme characterization supported the interconversions among these xanthine alkaloids in a complex metabolic network.

## Introduction

As one of the most popular non-alcoholic beverages in the world, tea is made from the leaves of tea plants [*Camellia sinensis* (L.) O. Kuntze] and owns diverse and pleasant flavors and health benefits. The tea flavors and health functions are mostly attributable to several characteristic secondary metabolites, including catechins, caffeine, theanine, and terpenoids in tea plant leaves (Chacko et al., 2010; Pinto, 2013; Yang and Zhang, 2019). Of them, caffeine (1,3,7-trimethylxanthine) is one key bioactive component present in many popular drinks worldwide, including tea and coffee. It could stimulate central nervous system, accelerate metabolic rate and be diuretic (Higdon and Frei, 2006). Because high-dosage intake of caffeine can cause health problems such as in sleeping and learning, thus an increasing number of consumers pursue caffeine-free or low-caffeine teas without changing their flavors. Thus, breeding of caffeine-free or low-caffeine tea plant varieties becomes one of the targets in tea plant breeding (Ashihara and Crozier, 2001; Mohanpuria et al., 2011; Deng et al., 2015).

Caffeine is the most abundant purine alkaloid in the section *Thea* plants and its content varies significantly from 1.5 to 60 mg/g in shoot tips (here including the apical buds and the first young leaves), averagely 35 mg/g in dry mass basis (Chen and Zhou, 2005; Yang et al., 2007; Jin J. Q. et al., 2014; Zhu et al., 2019). The divergent and convergent evolution of caffeine biosynthesis had been unveiled by genome sequencing of tea, coffee and cacao plants, and studies on other plants (Argout et al., 2011; Denoeud et al., 2014; Huang et al., 2016; Xia et al., 2017). The caffeine-specific pathway starts with a xanthosine as the common precursor, which is methylated into 7-methylxanthosine. 7-Methylxanthosine is hydrolyzed by a nucleosidase into 7-methylxanthine, which is then methylated at 3-*N* into theobromine (3,7-dimethylxanthine), and then further methylated at 1-*N* into caffeine (Kato et al., 1996; Deng and Ashihara, 2010). These three methylation steps are catalyzed by three *N*-methyltransferases (NMTs), including xanthosine methyltransferase (XMT), 7-methylxanthine methyltransferase (MXMT), and tea caffeine synthase (TCS), which all use *S*-adenosyl-L-methionine (SAM) as a methyl donor (Ashihara et al., 2017; Xia et al., 2017). At least five pathways provide xanthosine for caffeine biosynthesis: *de novo* route initiated from PRPP; AMP route by an AMP → IMP → XMP → xanthosine pathway; SAM cycle route by an adenosine (produced from SAM cycle) → adenine → AMP → IMP → XMP → xanthosine pathway; NAD route using the AMP (degraded from NAD by NAD pyrophosphatase) to synthesize xanthosine; and GMP route by a GMP → guanosine → xanthosine pathway (Ashihara et al., 2017).

Among these essential NMTs, tea caffeine synthase (TCS1) is a key enzyme involved in caffeine biosynthesis pathway by catalyzing 3-*N*- and 1-*N*-methylation of mono- and di-methylxanthines but not 7-*N*-methylation of xanthosine, and thus participating in the second and third methylation steps (Kato et al., 1999; Kato et al., 2000). PCS1 and ICS1 from *Camellia ptilophylla* and *C. irrawadiensis* respectively have only 3-*N*- but no 1-*N*-methyltransferase activity, which both belong to theobromine synthase (TS) or 7-methylxanthine methyltransferase (MXMT) converting 7-methylxanthine to theobromine (Yoneyama et al., 2006). Structural biology studies had revealed mechanisms underlying these NMT-catalyzed methylation reactions (Mizuno et al., 2003; Kato and Mizuno, 2004; Yoneyama et al., 2006). Four highly conserved regions including motif A, motif B’, motif C, and the YFFF-region exist in amino acids sequences of these alleles (Kato and Mizuno, 2004). The amino acid residue 225 of TCS1 plays a crucial role in substrate specificity (Yoneyama et al., 2006; Jin L. et al., 2014) and amino acid residue 269 is responsible for the difference in TCS activity and substrate recognition (Jin et al., 2016). TCS1 has six types of allelic variations (*TCS1a–f*) and *TCS1a* is the primary allele among section *Thea* plants, *ICS1* and *PCS1* are *TCS1b* and *TCS1c* respectively (Jin et al., 2016). Based on structure-activity relationship of TCS1, it was speculated that the large variations in *TCS1* alleles could be responsible for different caffeine contents in tea plant varieties (Jin et al., 2016). However, the genes of the *N*-methyltransferase that catalyzes 7-*N*-methylation of xanthosine and the nucleosidase that catalyzes the conversion of 7-methylxanthosine to 7-methylxanthine have not yet been cloned. Moreover, the exist of several tea plant varieties with extremely low caffeine but high theobromine contents strongly suggests that a major locus encoding 1-*N* MT (methyltransferase) enzyme present in tea plants and 1-*N* and 3-*N* MT loci were segregated. However, little is known about them (Ashihara et al., 2017; Zhao et al., 2020).

In comparison to extensive studies on caffeine biosynthesis, there are a few studies on caffeine catabolism in plants, which is not understood at either enzyme and molecular levels (Ashihara et al., 1995; Ashihara et al., 1997; Zheng et al., 2002; Silvarolla et al., 2004). Caffeine catabolism is extremely weak in tea leaves and most purine alkaloids are present in processed green teas. Based on several tracer experiments, caffeine degradation pathway is proposed: caffeine → theophylline → 3-methylxanthine → xanthine → uric acid → allantoin → allantoic acid → CO_2_ + NH_3_ + glyoxylic acid *via* urea and ureidoglycolic acid (Moffatt and Ashihara, 2002; Muñoz et al., 2006; Deng and Ashihara, 2010; Ashihara et al., 2017). A series of demethylation reactions could be critically required for the conversion of caffeine to theophylline, or theobromine, and xanthine (Ashihara et al., 1997; Ashihara and Crozier, 1999; Zhu et al., 2019). However, so far no gene encoding caffeine demethylase had ever been identified or characterized in tea plant (Zhao et al., 2020).

To gain more understanding of caffeine biosynthesis and catabolism, we conducted a series of feeding experiments with 15 mM caffeine or theophylline, or 3 mM theobromine, to detached tea plant shoot tips (including the apical buds and first young leaves) and roots of hydroponically cultured tea cuttage seedlings. We then also conducted degradation experiments with these shoot tips and roots absorbed and accumulated purine alkaloids to high concentrations. By monitoring purine alkaloid metabolites and transcriptomic analyses of shoot tips and roots fed with these purine alkaloids, we observed strong feedback inhibition on caffeine synthesis and inter-conversion between caffeine, theophylline, theobromine, and 3-methylxanthine. This work provides special insight on the understanding of caffeine biosynthesis and catabolism in tea plants. It also lays a foundation for further study of the catabolism of xanthine alkaloids at molecular levels.

## Materials and Methods

### Plant Materials, Growth Conditions, and Treatments

Shoot tips (including the apical buds and first young leaves) of tea plant (*Camellia sinensis* L., cv. Baihaozao) were plucked from the experimental field of Anhui Agricultural University at April in Hefei, China (E117°13’, N31°56’), two treatments to tea plant shoot tips were that feeding them with 15 mM caffeine and 15 mM theophylline respectively. After feeding for 48 h, shoot tips were rinsed, removed the surface water and transferred to a chamber with high humidity (90%–95%) under light at room temperature for examination of caffeine or theophylline degradation inside the shoot tips.

Two-year-old tea cuttage seedlings (*Camellia sinensis* L., cv. Shuchazao) attained from a tea garden of Tangchi, Hefei, China (E117°6’, N31°21’) were cultured by Shigeki Konishi (SK) solution in greenhouse with day and night temperatures of 26°C and relative humidity of 60%, the SK solution was updated once a week. Some tea plants were fed with 3 mM theobromine totally 72 h for examination of its metabolism in roots. Some tea plants were also fed with 15 mM caffeine and 15 mM theophylline independently, after feeding for 72 h, roots were cutted, rinsed, removed the surface water and transferred to a chamber with high humidity (90%–95%) under dark at room temperature, which was considered as the beginning of caffeine or theophylline degradation in roots.

Above shoot tips and roots were collected at different time points of feedings or degradations and immediately frozen in liquid nitrogen and kept at −80°C, which are the materials of metabolite profiling and transcriptomic analyses.

### Determinations and Identifications of Xanthine Alkaloids

Shoot tips and roots samples were ground to powder in liquid nitrogen with mortars and pestles. For determination of xanthine alkaloids, about 0.1 g (fresh weight) powder was extracted with 1.5 ml 80% (v) methanol in water and sonicated for 30 min at 12°C. All samples were mixed up and down every 10 min and centrifuged at 10,000 rpm for 10 min, then the supernatants were filtered through 0.22 µm membranes before UPLC analysis (Pan et al., 2019).

Xanthine alkaloids in the extracts were measured using an ACQUITY UPLC H-Class instrument (Waters, the United States) and separated on a ACQUITY UPLC BEH C18 Column (2.1 × 100 mm, 1.7 μm in particle size and 130 Å in pore size) at a flow rate of 0.3 ml/min at 35°C with 1 μl sample volume, the mobile phase was selected as 0.2% (v) acetic acid in water (A), and methanol (B), and the gradient elution was as follows: B 6% from 0 to 2 min, to 8% at 3.5 min, to 10% at 4.5 min, to 18% at 7.5 min, to 26% at 10.5 min, to 80% at 13.5 min and kept at 80% for 1 min, to 6% at 16 min and kept at 6% for 1 min, the detection wavelength was set to 278 nm (Li et al., 2017; Pan et al., 2019). Xanthine alkaloids were identified by using a high resolution quadrupole time-of-flight mass spectrometry (QTOF MS) on the positive-ion mode, the Agilent 1290 UPLC was interfaced with an Agilent 6545 Q-TOF LC/MS system equipped with electrospray ionization source (ESI). The combination of full scan MS and targeted MS/MS data acquisition enable confident identification (Huang et al., 2016).

### RNA-Seq Analysis

Shoot tip and root samples were also used for RNA-Seq analyses. Total RNAs were extracted by using RNA extraction and purification kit (Takara, Beijing, China) according to the manufacturer’s protocols. RNA integrity and concentration were detected by Agilent 2100 Bioanalyzer (Agilent, USA). cDNA libraries construction and RNA-Seq performed on an Illumina HiSeq 4000 sequencing platform were carried out professionally by BGI (Shenzhen, China).

Hierarchical indexing for spliced alignment of transcripts (HISAT2, version 2.1.0, http://www.ccb.jhu.edu/software/hisat) was used to map the clean reads to the tea plant genome (Wei et al., 2018) and the average mapping rate was 82.37%, clean reads were also mapped to reference tea plant genome (Wei et al., 2018) using Bowtie2 and the average mapping rate was 66.63%. The novel transcripts were attained after using StringTie, Cuffmerge, and Cuffcompare successively, then the coding potential calculator (CPC, version 0.9-r2, http://cpc.cbi.pku.edu.cn) was used to predict the protein-coding potential of the novel transcripts.

Gene expression levels of total samples were calculated by using RSEM package (version 1.2.8). Differentially expressed gene (DEG) was analyzed by using DEGseq method based on Poisson distribution (Wang et al., 2010). In order to improve the accuracy of DEGs, genes with fold change of more than two times and adjusted P-values less than 0.001 were screened as significantly differentially expressed genes. DEGs were then annotated into different GO (Gene ontology) terms and different KEGG (Kyoto encyclopedia of genes and genomes) pathways, the phyper function in R software was used for GO and KEGG pathway enrichment analyses. Q-value, that is, P-value after FDR (False discovery rate) calibration was calculated and Q-value less than 0.05 was defined as enriched significantly.

### Quantitative Real-Time PCR (qRT-PCR) Analysis

QRT-PCR was performed to validate the accuracy of transcriptomic data. Around 1 μg extracted total RNA was used to synthesize first-strand cDNA with the PrimeScript^™^ RT Master Mix kit (Takara, Dalian, China). QRT-PCR was performed in a 20 μl volume containing 7.6 μl ddH_2_O, 0.8 μl forward primer, 0.8 μl reverse primer, 0.8 μl cDNA, and 10 μl TB Green^®^ *Premix Ex Taq*^™^ II (Takara, Dalian, China). The tea *β-actin* gene was used as an internal reference gene (GenBank: HQ420251.1), its forward primer and reverse primer (both from 5’ end to 3’ end) were designed as GCCATCTTTGATTGGAATGG and GGTGCCACAACCTTGATCTT respectively. Other qRT-PCR primers were designed by Primer Premier 6.0 software (PREMIER Biosoft Company, http://www.premierbiosoft.com/index.html) and listed in **Supplementary Table S1**. The qRT-PCR was carried out in the CFX96^™^ Real-Time System (Bio-Rad, USA). The cycling profile was 95°C, 30 s; 40 cycles of 95°C, 5 s and 60°C, 30 s. Relative gene expression was calculated with the 2^-ΔΔC^_T_ method (Livak and Schmittgen, 2001) from three technical replicates.

### Identification of Caffeine Biosynthetic Genes in the Camellia sinensis Genome

BLASTP against the *Camellia sinensis* proteome dataset obtained from the genome sequence (Tea plant genome (Wei et al., 2018); The Tea Plant Information Archive-TPIA public database: http://teaplant.org/) was used to search for all structural genes involved in caffeine biosynthesis pathways with characterised homologue protein sequences from other plants as queries. Unrooted phylogenetic trees were constructed following the Neighbour-Joining method involving 500 replicates with the bootstrap test in MEGA 7.0 (**Supplementary Figure S1**). Gene expression of identified caffeine biosynthesis genes in eight tissues (apical bud; young, mature and old leaves; root; stem; flower; and fruit: **Supplementary Table S2**) was retrieved from the microarray-based transcriptome tea data for the cultivar Shuchazao [Tea plant genome (Wei et al., 2018), TPIA public database: http://teaplant.org/].

Among 49 *MT* (methyltransferase) genes used for building the phylogenetic tree (**Supplementary Figure S2**), eight tea caffeine synthase genes (*TCS-1* to *TCS-8*) from tea plant genome CSS (Wei et al., 2018), six alleles of *TCS1* (*TCS1a*, *b*, *c*, *d*, *e*, *f*) were from various tea plant varieties (Jin et al., 2016), six *MXMT-2* to *MXMT-7* genes from tea plant genome (Wei et al., 2018), 13 xanthosine methyltransferase (*XMT-1* to *XMT-13*) genes from tea plant genome (Wei et al., 2018), four indole-3-acetate *O*-methyltransferase genes from tea plant genome (Wei et al., 2018), which are annotated according to KEGG database (TEA014703.1, TEA017731.1, TEA031962.1, and TEA032424.1), one *Coffea arabica* xanthosine methyltransferase (*CaXMT1*), and 11 *Camellia MT* genes published previously.

### Heterologous Expression of Two NMT (N-Methyltransferase) Genes in Escherichia coli

The open reading frames (ORFs) of *TCS-4* and *TCS-8* were cloned from the cDNA of tea plant (*Camellia sinensis* L., cv. Shuchazao) buds and ligated into pGEX-4T-1 vector which could induce the synthesis of GST (Glutathione *S*-transferase)-fusion proteins. The forward and reverse primers were designed with an *Eco*RI and a *Not*I restriction site at their 5’ ends respectively and are shown in **Supplementary Table S1**. The ligations of these two genes with pGEX-4T-1 vector were performed by a T4-ligase system (Takara, Dalian, China) and confirmed by sequencing in General Biosystems (Anhui, China). Recombinant plasmids were transferred into the *E. coli Trans*etta (DE3) chemically competent cell (Transgen, Beijing, China). The positive clones were chosen and shaken in 3 ml LB (Luria-Bertani) medium with 50 μg/ml ampicillin for 12 h at 37°C, then 500 μl *E. coli* cultures were added into 50 ml LB medium with 50 μg/ml ampicillin and shaken at 200 rpm and 37°C till OD_600_ value were between 0.6 and 0.8, then 0.5 mM final concentration IPTG (Isopropyl-β-D-thiogalactopyranoside) was added to induce the expression of these two genes at 16°C, 200 rpm. For SDS-PAGE analysis, 5 μg crude proteins from uninduced cells and induced cells were boiled with loading buffer and subjected to 10% SDS-PAGE.

### Enzyme Activity Assay

After induced at 16°C for 4 h, eight substrates (xanthosine, xanthine, 7-methylxanthine, 3-methylxanthine, 1-methylxanthine, theobromine, paraxanthine, theophylline) were added to *E. coli* cultures individually, the concentrations of the substrates are all 1 mM. Then the cultures were incubated continually at 16°C for 20 h, after that they were incubated at 30°C for appropriate time. *E. coli* cultures were sampled at various time points and centrifuged at 10,000 rpm for 10 min, and supernatants were filtered with 0.22 μm membranes before the detection of xanthine alkaloids using above-mentioned UPLC method.

### Bioinformatic Analysis

Heatmaps were constructed with the R package. Multiple sequences alignment analysis was performed using the ClustalW program in the software MEGA 7.0. Phylogenetic trees were constructed for caffeine synthetic enzymes with homologue protein sequences using the NJ (Neighbor-Joining) statistical method in MEGA 7.0, with parameters of bootstrap method (500 replications), p-distance model, Uniform rates, and Partial deletion (50% site coverage cutoff).

### Statistical Analysis

All the data presented in this study were obtained from at least three independent biological replicates, including xanthine alkaloids feeding experiments, metabolite profiling, RNA-Seq, and qRT-PCR analysis. Statistical analyses were conducted using the Minitab 16.0 statistical software package (Minitab Inc., Coventry, UK). Data were analyzed by one-way analysis of variance (ANOVA) and a Fisher’s least significant difference (LSD) test at the 5% level.

## Results

### Effects of Caffeine Feeding on Xanthine Alkaloids in Tea Plant Shoot Tips and Roots

**Figure 1:**
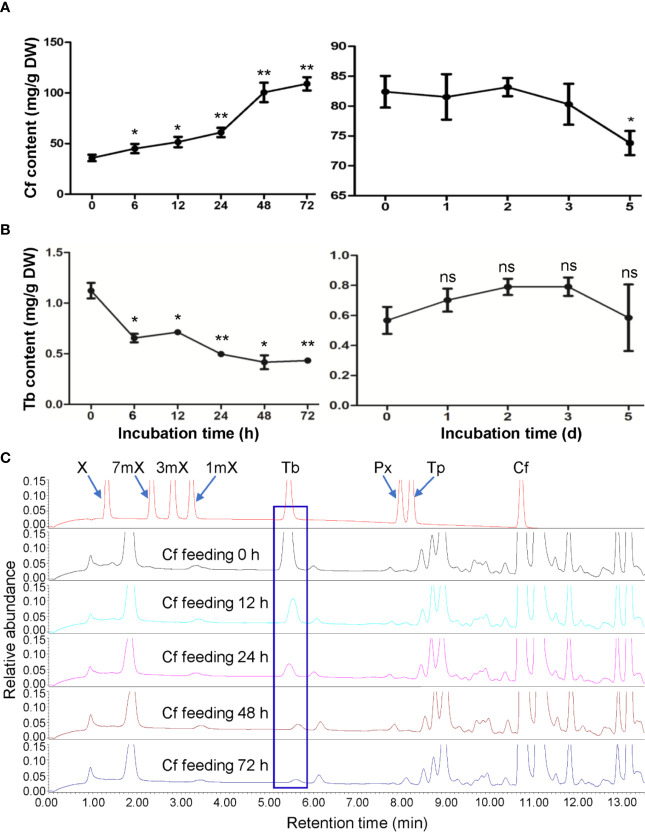
Effects of caffeine feeding to detached tea plant shoot tips on xanthine alkaloids. **(A)** Caffeine (Cf) content in shoot tips during feeding (left panel) and degradation (right panel). **(B)** Theobromine (Tb) content in shoot tips during feeding (left panel) and degradation (right panel). **(C)** UPLC traces for changed xanthine alkaloids in shoot tips during Cf feeding. X, xanthine; 7mX, 7-methylxanthine; 3mX, 3-methylxanthine; 1mX, 1-methylxanthine; Px, paraxanthine; Tp, theophylline. Data are presented as means ± SD from at least three independent repeats. Two-tailed Student’s *t*-tests were performed to compare data at each time point against the control (0 h or 0 day). Significant differences are labeled with asterisks (*P < 0.05, **P < 0.01), ns, no significance. Representative UPLC traces are shown.

To understand caffeine catabolism in tea plant, we fed the fresh detached tea plant shoot tips (including apical buds and the first young leaves) with Shigeki Konishi (SK) solution containing 15 mM caffeine. These shoot tips from tea plants (*Camellia sinensis* L., cv. Baihaozao) contain about 35.87 mg/g caffeine (on dry mass basis). After 72 h of feeding, the caffeine contents in shoot tips increased to 109 mg/g, indicating the caffeine was taken up by shoot tips (**Figure 1A**). We then transferred the rinsed shoot tips from caffeine-feeding solution to a chamber with high humidity (90%–95%) under light at room temperature for examination of caffeine degradation inside the shoot tips. Caffeine content in these shoot tips slightly decreased from 82.42 to 73.83 mg/g during the degradation period (**Figure 1A**), suggesting that only about 10.4% caffeine was degraded in tea plant shoot tips. Simultaneously, theobromine contents also decreased significantly upon caffeine feeding to shoot tips for 6 h (**Figure 1B**) and continued the decrease in caffeine-fed shoot tips for 12 to 72 h (**Figures 1B, C**). Because theobromine can be converted into caffeine as the last step of caffeine biosynthesis, decreases in the major precursor for caffeine biosynthesis may also reduce the caffeine biosynthesis rate significantly, suggesting that there is a feedback loop for regulation of caffeine synthesis in tea plants.

**Figure 2:**
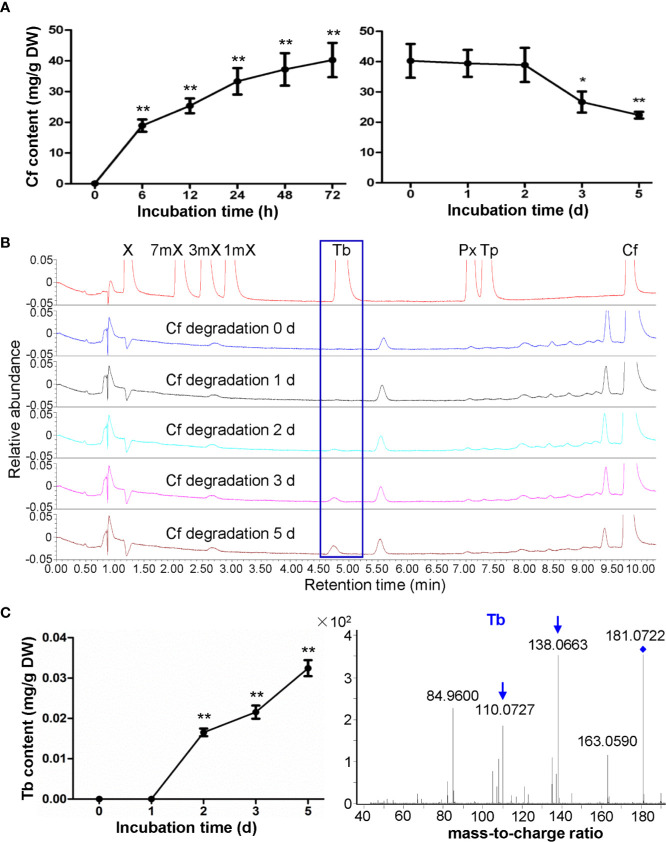
Effects of caffeine feeding to tea plant roots on xanthine alkaloids. **(A)** Caffeine (Cf) content in roots during feeding (left panel) and degradation (right panel). **(B)** UPLC traces for changed xanthine alkaloids in roots during Cf degradation. X, xanthine; 7mX, 7-methylxanthine; 3mX, 3-methylxanthine; 1mX, 1-methylxanthine; Tb, theobromine; Px, paraxanthine; Tp, theophylline. **(C)** Tb content in roots during Cf degradation (left panel) and identification of Tb from roots by using LC-MS/MS (right panel). Data are presented as means ± SD from at least three independent repeats. Two-tailed Student’s *t*-tests were performed to compare data at each time point against the control (0 h or 0 day). Significant differences are labeled with asterisks (*P < 0.05, **P < 0.01). Representative UPLC traces and LC-MS spectrometry are shown.

There is only trace amount of caffeine in the roots of hydroponically growing tea cuttage seedlings. However, upon caffeine feeding for 72 h, the caffeine contents in roots rapidly increased to 40.27 mg/g (dry mass basis) (**Figure 2A**). When transferred these roots to another closed chamber at high humidity (90%–95%) for examination of degradation of caffeine inside the roots, it was observed that caffeine contents in roots decreased significantly to 26.67 mg/g after incubation for 3 days (**Figure 2A**). The final caffeine contents could decrease to 22.35 mg/g in dry roots after caffeine degradation for 5 days (**Figure 2A**). In total, about 44.5% caffeine was degraded in roots after 5 days of degradation. Compared with the results in shoot tips, caffeine degradation rate in tea plant roots was significantly higher than that in shoot tips.

While very low level of theobromine can be detected in regular tea plant roots, UPLC traces revealed that theobromine clearly emerged in tea plant roots fed with caffeine and then used for caffeine degradation for 2 days, and theobromine contents increased gradually from then on (**Figures 2B, C**). Since we did not detect theophylline in these root samples, it was suggested that caffeine might be degraded back to theobromine in roots, unlike caffeine degradation into theophylline in tea plant roots. LC-MS/MS analysis of these samples also proved that this compound was theobromine for the detection of daughter ion of 138.0663 and 110.0727 (**Figure 2C**).

### Effects of Theobromine Feeding on Xanthine Alkaloids in Tea Plant Roots

**Figure 3:**
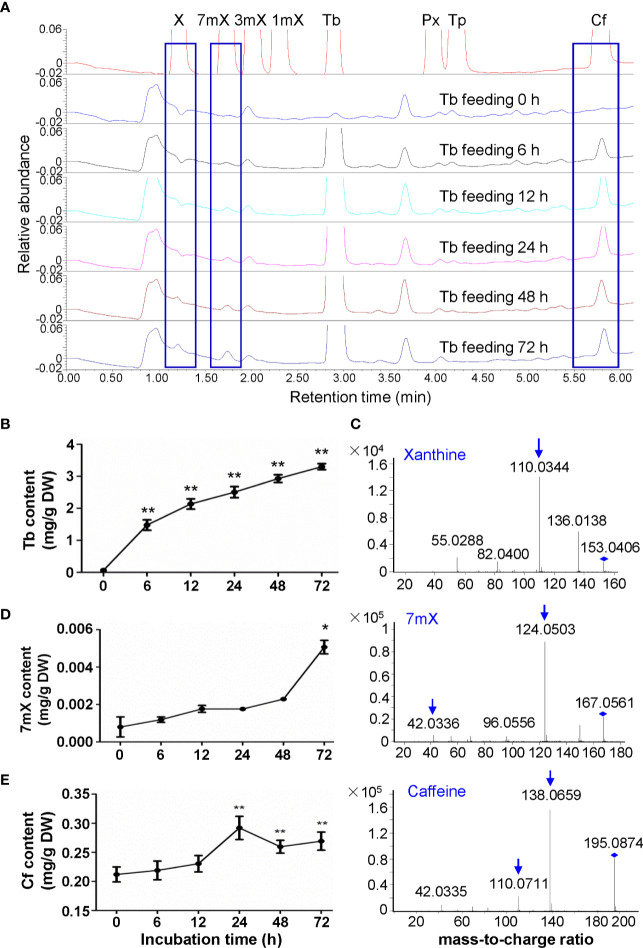
Effects of theobromine feeding to tea plant roots on xanthine alkaloids. **(A)** UPLC traces for changed xanthine alkaloids in roots during theobromine (Tb) feeding. X, xanthine; 7mX, 7-methylxanthine; 3mX, 3-methylxanthine; 1mX, 1-methylxanthine; Px, paraxanthine; Tp, theophylline; Cf, caffeine. **(B)** Tb content in roots during Tb feeding. **(C)** Identification of X from roots by using LC-MS/MS. **(D)** 7mX content in roots after Tb feeding (left panel) and identification of 7mX from roots by using LC-MS/MS (right panel). **(E)** Cf content in roots after Tb feeding (left panel) and identification of Cf from roots by using LC-MS/MS (right panel). Data are presented as means ± SD from at least three independent repeats. Two-tailed Student’s *t*-tests were performed to compare data at each time point against the control (0 h). Significant differences are labeled with asterisks (*P < 0.05, **P < 0.01). Representative UPLC traces and LC-MS spectrometry are shown.

We then fed the tea plant roots with theobromine for examination of its biodegradation in roots. Usually tea plant roots contain much less theobromine as mentioned above. However, theobromine contents increased to 3.30 mg/g after theobromine feeding for 72 h (**Figures 3A, B**). Interestingly, xanthine (X) was also detected in theobromine-fed roots by using LC-MS/MS with a daughter ion peak of 110.0344 (**Figure 3C**). Meanwhile, we observed that 7-methylxanthine (7mX) content increased significantly from a very low basic level (0.0008 mg/g) to 0.0051 mg/g after theobromine feeding for 72 h. We confirmed the 7mX identified by LC-MS/MS for the detection of daughter ion of 124.0503 and 42.0336 (**Figure 3D**). These results suggested that theobromine might be degraded to 7mX and X successively in tea plant roots. Upon theobromine feeding, caffeine content increased significantly with theobromine absorption into the roots for 24 h, as compared with the control roots (**Figure 3E**). This suggested that theobromine absorbed into the tea plant roots also entered into the caffeine biosynthesis pathway as a precursor during theobromine feeding. Theobromine feeding not only supplied precursors for caffeine synthesis, but also promoted the excessive theobromine to be demethylated by unknown enzymes to other xanthine alkaloids during theobromine feeding to tea plant roots.

### Effects of Theophylline Feeding on Xanthine Alkaloids in Tea Plant Shoot Tips and Roots

**Figure 4:**
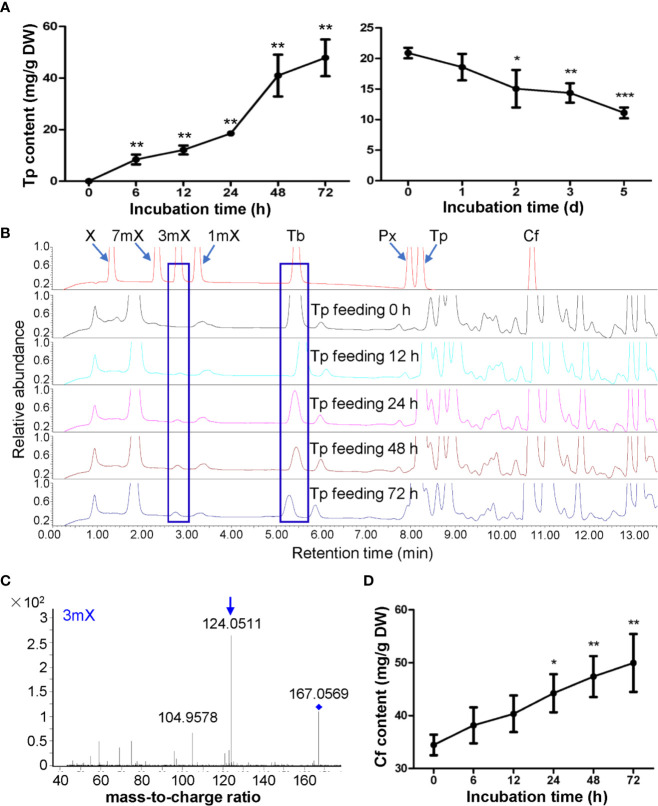
Effects of theophylline feeding to detached tea plant shoot tips on xanthine alkaloids. **(A)** Theophylline (Tp) content in shoot tips during feeding (left panel) and degradation (right panel). **(B)** UPLC traces for changed xanthine alkaloids in shoot tips during Tp feeding. X, xanthine; 7mX, 7-methylxanthine; 3mX, 3-methylxanthine; 1mX, 1-methylxanthine; Tb, theobromine; Px, paraxanthine; Cf, caffeine. **(C)** Identification of 3mX from shoot tips by using LC-MS/MS. **(D)** Changes in Cf content in shoot tips after fed with Tp for different time. Data are presented as means ± SD from at least three independent repeats. Two-tailed Student’s *t*-tests were performed to compare data at each time point against the control (0 h or 0 day). Significant differences are labeled with asterisks (*P < 0.05, **P < 0.01). Representative UPLC traces and LC-MS spectrometry are shown.

Theophylline in shoot tips of tea plant (*Camellia sinensis* L., cv. Baihaozao) was almost not detected, however, it increased to 47.85 mg/g after being fed with theophylline for 72 h (**Figure 4A**), suggesting that incubation of detached tea plant shoot tips in a solution containing theophylline could promote theophylline absorption into shoot tips. However, when removing these shoot tips from solutions and incubating them in Petridis chambers with 90%–95% humidity under light for degradation assay, the theophylline contents decreased significantly from 20.90 mg/g at day 0 to 15.06 mg/g at day 2, and then decreased continually to 11.11 mg/g at day 5 (**Figure 4A**). About 46.8% of total theophylline absorbed into the shoot tips was degraded. UPLC traces indicated that 3-methylxanthine (3mX) emerged after theophylline feeding for 12 h and had slightly increased from then on (**Figure 4B**). We confirmed the 3mX by using LC-MS/MS, which showed the daughter ion of 124.0511 was detected (**Figure 4C**). Interestingly, caffeine content in shoot tips fed with theophylline also increased gradually during theophylline feeding (**Figure 4D**), suggesting that theophylline may act as another precursor, besides the well-known theobromine, for synthesis of caffeine in tea plant shoot tips. It could also be possible that 3mX may be recycled back to theobromine, which then be used by TCS for caffeine synthesis.

**Figure 5:**
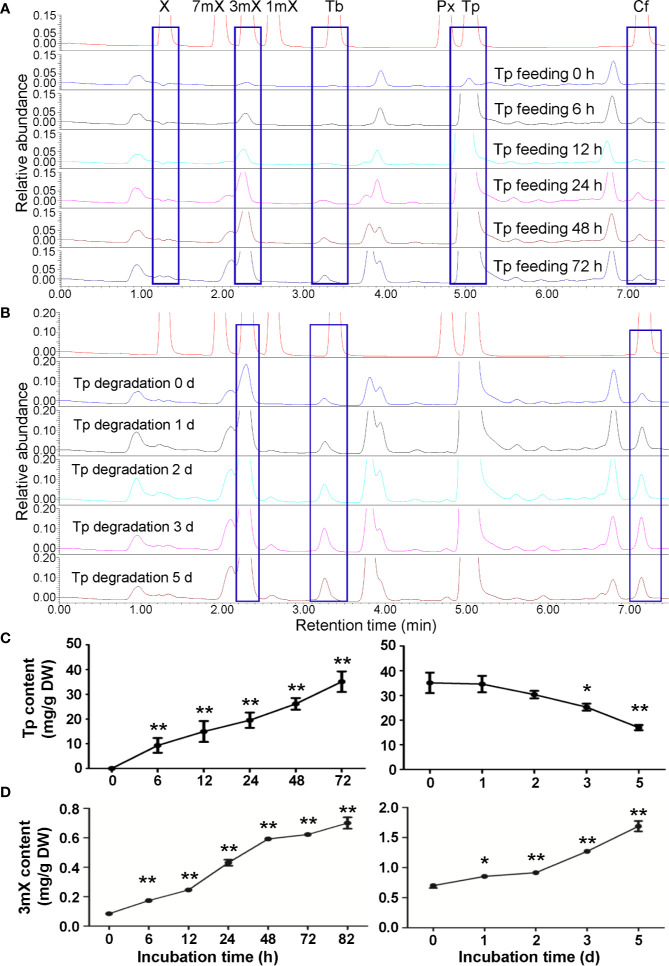
Effects of theophylline feeding to tea plant roots on xanthine alkaloids. **(A)** UPLC traces for changed xanthine alkaloids in roots during theophylline (Tp) feeding. X, xanthine; 7mX, 7-methylxanthine; 3mX, 3-methylxanthine; 1mX, 1-methylxanthine; Tb, theobromine; Px, paraxanthine; Cf, caffeine. **(B)** UPLC traces for changed xanthine alkaloids in roots during Tp degradation. **(C)** Tp content in roots during feeding (left panel) and degradation (right panel). **(D)** 3mX content in roots during feeding (left panel) and degradation (right panel). Data are presented as means ± SD from at least three independent repeats. Two-tailed Student’s *t*-tests were performed to compare data at each time point against the control (0 h or 0 day). Significant differences are labeled with asterisks (*P < 0.05, **P < 0.01). Representative UPLC traces and LC-MS spectrometry are shown.

Theophylline was also rapidly absorbed into roots when fed with theophylline in solutions. UPLC traces and 3mX quantification revealed that the content of 3mX in roots increased gradually during theophylline feeding and degradation (**Figures 5A, B, D**). Interestingly, both theobromine and caffeine contents increased gradually during theophylline feeding, as compared with the control roots. These two compounds continued to increase during degradation period (**Figures 5A, B**), which suggested that theophylline might participate in caffeine biosynthesis through a theophylline → 3-methylxanthine → theobromine → caffeine pathway. This proposed pathway is also consistent with the findings on trace amounts of 3mX generated in shoot tips fed with theophylline, and on a significant increase of caffeine content in shoot tips fed with theophylline for 24 h (**Figures 4B, D**). Theophylline contents increased to 35.10 mg/g after 72 h of theophylline feeding. Theophylline contents in roots were then decreased significantly to 25.26 mg/g after roots were removed from feeding solution and incubated in closed high humidity (90%–95%) chamber for theophylline degradation for 3 days. Finally, theophylline contents decreased to 16.97 mg/g in dry roots after 5 days of theophylline degradation (**Figure 5C**). About 51.7% theophylline was degraded in tea plant roots. Compared with that in tea plant shoot tips, theophylline degradation rate in tea plant roots was basically similar.

Therefore, above proposed pathway from theophylline → 3-methylxanthine → theobromine → caffeine might present in tea plants. In addition, caffeine degradation rate was significantly slower than theophylline in tea plant shoot tips, suggesting that the conversion of caffeine to theophylline is the rate-limiting step in caffeine catabolism pathway, which was consistent with previous study (Ashihara et al., 1997).

### Phylogenetic Identifications and Expression Patterns of Genes Involved in Purine Alkaloid Biosynthesis

**Figure 6:**
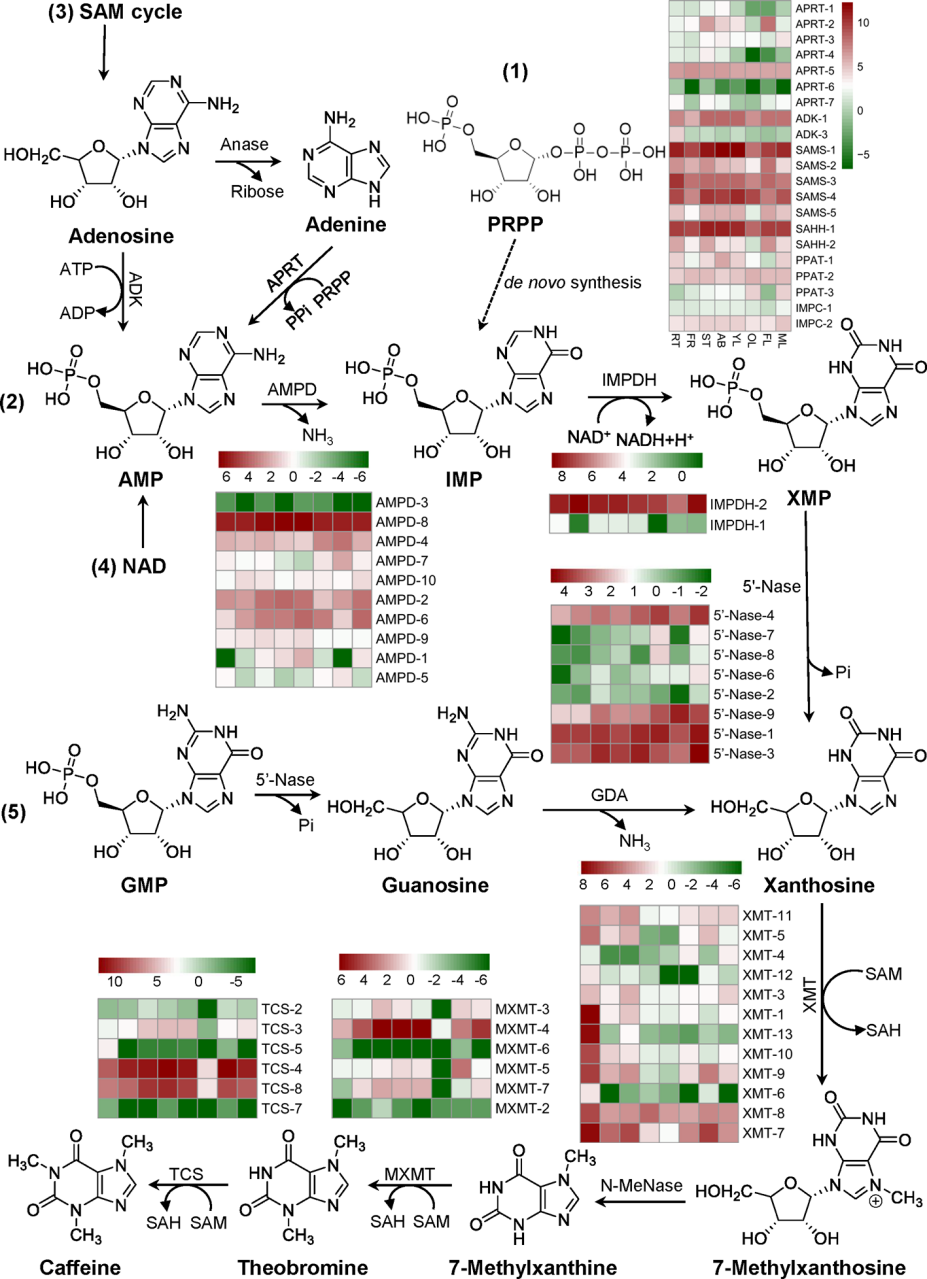
Identification and expression profiles of genes involved in caffeine biosynthesis. Five pathways towards caffeine synthesis: 1) *De novo* route, 2) AMP route, 3) SAM cycle route, 4) NAD route, and 5) GMP route were listed. Expression profiles of major genes in eight tissues: apical buds (AB), young leaves (YL), mature leaves (ML), old leaves (OL), young stems (ST), tender roots (RT), flowers (FL), and young fruits (FR) were evaluated using FPKM. Abbreviations: amidophosphoribosyltransferase/PRPP amidotransferase (PPAT) which catalyzes the conversion of PRPP to PRA in the first step of *de novo* route; IMP cyclohydrolase (IMPC) which catalyzes the conversion of FAICAR to IMP in *de novo* route; AMP deaminase (AMPD); IMP dehydrogenase (IMPDH); 5’-nucleotidase (5’-Nase); *S*-adenosyl-L-methionine synthase (SAMS); *S*-adenosyl-L-homocysteine hydrolase (SAHH); adenosine kinase (ADK); adenosine nucleosidase (Anase); adenine phosphoribosyltransferase (APRT); guanosine deaminase (GDA); xanthosine methyltransferase/7-methylxanthosine synthase (XMT); *N*-methylnucleosidase (*N*-MeNase); methylxanthine methyltransferase/theobromine synthase (MXMT); tea caffeine synthase/3,7-dimethylxanthine methyltransferase (TCS). PRPP, 5-phosphoribosyl-1-pyrophosphate; PRA, 5-phosphoribosyl amine; FAICAR, 5-formamidoimidazole-4-carboxyamide ribonucleotide.

The tissue expression patterns of genes involved in *de novo* route initiated from PRPP; AMP route from AMP to xanthosine pathway; SAM cycle route from an adenosine to xanthosine pathway; NAD route from AMP degraded from NAD by NAD pyrophosphatase xanthosine; and GMP route from a GMP to xanthosine pathway were identified from tea plant genome (**Supplementary Figure S1**). Three methylation steps catalyzed by three *N*-methyltransferases (NMTs), including xanthosine methyltransferases (XMT), 7-methylxanthine methyltransferases (MXMT), and tea caffeine synthases (TCS), which all use *S*-adenosyl-L-methionine (SAM) as a methyl donor, were also identified in tea genome (**Supplementary Figure S1**). The evolutionary relationship of 49 *MT* genes was performed by Neighbor-Joining distance analysis in MEGA 7.0. All the GenBank accession numbers of genes in the phylogenetic tree are summarized in **Supplementary Table S3**. Except *MXMT-7* (TEA030024.1) and *ICS2* (BAE79731.1), the other 47 *MT* genes were simply classified to five groups (I, II, III, IV, and V) (**Supplementary Figure S2**). Expression patterns of genes involved in caffeine biosynthesis were shown on **Figure 6**. The putative genes encoding enzymes involved in caffeine degradation were also identified from the tea plant genome. *Camellia sinensis* xanthine dehydrogenase *(CsXDH)*, urate oxidase *(CsUOX)*, allantoinase *(CsALN)* and urease *(CsURE)* are all multi-gene families (**Supplementary Figure S3**, **Table S4**).

### RNA-Seq Analysis of Tissues Fed With Caffeine and Theophylline

To investigate the molecular mechanism of xanthine alkaloids variations during feeding and degradation of caffeine and theophylline, 12 treatment samples of shoot tips and roots at different time points were sequenced using an Illumina HiSeq 4000 platform. These RNA-Seq generated about 6.83 Gb data averagely for each sample. About 64,667 expressed genes were detected, among them 33,352 genes have known functional annotations and 31,315 are predicted novel genes. Among 192,692 of novel transcripts identified from transcriptome, 42,334 belong to new alternative splicing subtypes of known protein-coding genes, 31,535 belong to transcripts of new protein-coding genes and the rest 118,823 belong to long non-coding RNA. Q20 of clean reads ranges from 96.3% to 98.36% and Q30 from 87.84% to 91.57% among those sequencing samples, which indicated that the obtained transcriptomic data were high-quality and qualified for further analysis.

Analyses of differentially expressed genes (DEGs) in the shoot tips and roots fed with caffeine or theophylline with their controls revealed a large number of genes were altered in their expression levels significantly (**Supplementary Figure S4**). Comparison of roots fed with caffeine for 48 h (as control) and caffeine degradation in roots for 3 days followed feeding (Cf_R_F48h-vs-Cf_R_D3d) and the comparison of roots fed with theophylline for 24 h with its control (0 h feeding) (R_F0h-vs-Tp_R_F24h) displayed almost similar numbers of DEGs, 28298 and 28772, respectively, which are larger than other comparisons. The comparisons with the lowest DEG numbers were in shoot tips fed with caffeine for 48 h with control (feeding for 0 h) (B_F0h-vs-Cf_B_F48h), which had only 9096 DEGs, and in shoot tips fed with theophylline for 48 h with its control (feeding for 0 h) (B_F0h-vs-Tp_B_F48h), which had only 8986 DEGs. Obviously, these feedings with high concentrations of caffeine or theophylline had induced much more drastic changes in global gene expression in tea plant roots than these did in tea plant shoot tips, suggesting that tea plant shoot tips are more physiologically adaptive to higher concentrations of these purine alkaloids than roots. This could also explain why caffeine or theophylline degradation is more active in roots than in shoot tips.

### DEGs Involved in Caffeine Biosynthesis in Purine Alkaloids Fed Shoot Tips and Roots

Since tea plant shoot tips or roots fed with caffeine, theobromine, or theophylline had drastically changed the xanthine alkaloid biosynthesis or biodegradation, we then conducted RNA-Seq on these samples to examine the related gene expression. We examined and identified caffeine or theophylline on gene expressions involved in caffeine biosynthesis pathway. 7-Methylxanthine methyltransferase (MXMT) and tea caffeine synthase (TCS) catalyze the last two *N*-methylations in caffeine biosynthesis pathway respectively. They are considered as two key enzymes in the pathway and extensively studied compared with other enzymes involved in this pathway.

**Figure 7:**
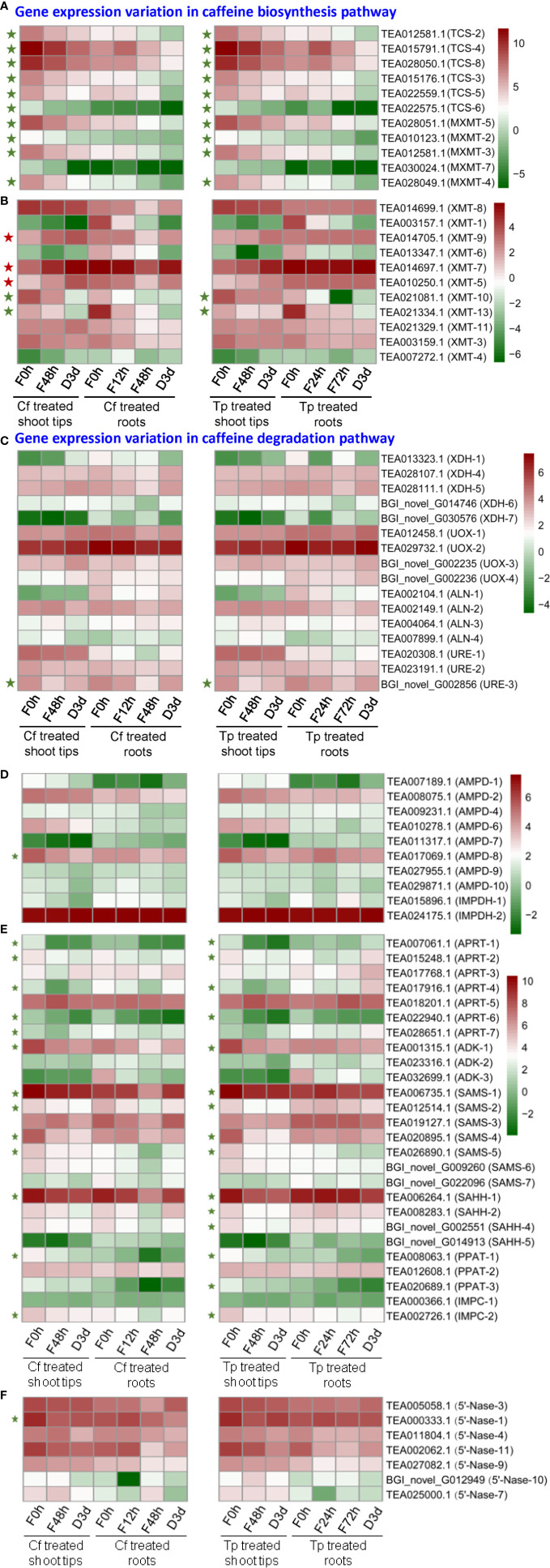
Differential expression of genes (DEGs) involved in caffeine biosynthesis **(A, B, D–F)** and degradation pathway **(C)** in alkaloid-fed shoot tips and roots. Green and red pentagrams indicate down-regulated DEGs and up-regulated DEGs respectively in the comparison of caffeine or theophylline feeding for 48 h and its control (caffeine or theophylline feeding for 0 h).

Expression levels of *MXMTs* and *TCSs* in tea plant shoot tips are much higher than in roots. Except for *MXMT-7* (TEA030024.1), the transcripts of all other *MXMT*s and *TCS*s in shoot tips were significantly down-regulated upon caffeine or theophylline feeding for 48 h. These transcripts of *MXMTs* and *TCSs* continuously decreased in caffeine- or theophylline-fed shoot tips after set for caffeine or theophylline degradation for 3 days (**Figure 7A**). Except for *TCS-6* (TEA022575.1) which was not changed drastically, other five *TCS* genes were all down-regulated significantly in caffeine degradation shoot tips for 3 days as compared with caffeine degradation for 0 day. These results indicated that feeding with high concentrations of caffeine or theophylline could feedback repress the expression of the most *MXMT*s and *TCS*s. Thus from both metabolite profiling and two key gene expression patterns, it is posited that caffeine or theophylline feeding inhibited caffeine synthesis through a negative feedback in tea plant shoot tips.

*TCS-4* (TEA015791.1) transcript level increased significantly by about 2.5-fold and decreased by about 2-fold in tea plant roots after fed with caffeine for 12 and 48 h, respectively, as compared with that of control (feeding for 0 h) (**Figure 7A**). *TCS-4* and *TCS-8* (TEA028050.1) are the most abundant *TCS* genes in tea plant shoot tips, both genes were expressed by 3.81 and 3.04 times lower, respectively, in caffeine-fed shoot tips for 48 h than in normal shoot tips. In addition, these two major *TCS* genes were also down-regulated in tea plant shoot tips fed with theophylline for 48 h, by 3.19 and 2.61 times, respectively, compared with these in non-fed control shoot tips. These data indicated that caffeine feeding to shoot tips drastically repressed major *TCS* genes more severely than theophylline feeding did (**Figure 7A**).

*MXMT-2* (TEA010123.1), *MXMT-3* (TEA012581.1), *MXMT-4* (TEA028049.1), and *MXMT-5* (TEA028051.1) were all down-regulated significantly in shoot tips by caffeine feeding for 48 h compared with non-fed control (feeding for 0 h), also in caffeine degradation shoot tips for 3 days as compared with caffeine degradation for 0 day. Except for *MXMT-2*, which was not changed significantly in theophylline degradation in shoot tips for 3 days compared with theophylline feeding for 48 h, these observations were also true when caffeine was replaced with theophylline at the same concentration for feeding or degradation experiments (**Figure 7A**). Xanthosine methyltransferase (XMT) catalyzes the conversion of xanthosine to 7-methylxanthosine, which is the first methylation step in caffeine biosynthesis. *XMT-10* (TEA021081.1) and *XMT-13* (TEA021334.1) were down-regulated significantly after caffeine or theophylline feeding for 48 h in shoot tips compared with feeding for 0 h. By contrast, *XMT-5* (TEA010250.1), *XMT-7* (TEA014697.1), and *XMT-9* (TEA014705.1) were significantly up-regulated (**Figure 7B**). The expression variations in genes involved in caffeine degradation pathway was shown in **Figure 7C**. The transcripts of *CsXDH-5* and *CsUOX1, 2, 3, 4* homologs displayed increases in caffeine degradation at day 3; while these of *CsALNs* and *CsUREs* were in mixed trends (**Figure 7C**). AMP deaminase (AMPD) catalyzes the conversion of AMP to IMP. *AMPD-8* (TEA017069.1) was downregulated significantly after caffeine feeding for 48 h compared with feeding for 0 h, and its expression in shoot tips fed with caffeine for 48 h was 1.7 times higher than that in caffeine degradation for 3 days (**Figure 7D**). IMP dehydrogenase (IMPDH) catalyzes the conversion of IMP to XMP. Only two genes are annotated to IMPDH in tea plant genome, that are, *IMPDH-1* (TEA015896.1) and *IMPDH-2* (TEA024175.1), and IMPDH-2 expression is significantly higher than IMPDH-1 in control tea plant shoot tips and roots. IMPDH-1 expression in shoot tips decreased gradually during caffeine or theophylline feeding and degradation, while IMPDH-2 expression increased a little at caffeine or theophylline degradation for 3 days compared with caffeine or theophylline feeding for 48 h (**Figure 7D**).

SAM (*S*-adenosyl-L-methionine) is known as the important methyl donor during the methylations of caffeine synthesis, and SAM synthetase (SAMS) catalyzes the conversion of methionine to SAM. *SAMS-1* (TEA006735.1), *SAMS-2* (TEA012514.1), *SAMS-4* (TEA020895.1) and *SAMS-5* (TEA026890.1) expressions all decreased significantly after caffeine or theophylline feeding for 48 h in shoot tips compared with the control (caffeine or theophylline feeding for 0 h) (**Figure 7E**). *S*-adenosyl-L-homocysteine hydrolase (SAHH) catalyzes the conversion of SAH (*S*-adenosyl-L-homocysteine) to homocysteine in SAM cycle pathway, and releasing adenosine in this step which may participate in caffeine biosynthesis as is referred to in ‘introduction’ part. *SAHH-1* (TEA006264.1), *SAHH-2* (TEA008283.1), and *SAHH-4* (BGI_novel_G002551) expressions decreased significantly after theophylline feeding for 48 h (**Figure 7E**).

Adenosine kinase (ADK) catalyzes the conversion of adenosine to AMP. *ADK-1* (TEA001315.1) expression is the most abundant in control shoot tips and it decreased significantly after caffeine or theophylline feeding for 48 h (**Figure 7E**). 5’-Nucleotidase (5’-Nase) plays a role in the removing of 5’-phosphate group of a nucleotide, like catalyzing the conversion of XMP to xanthosine, and the latter compound is known as the initial methyl acceptor for the main caffeine biosynthesis pathway in tea plants. Gene expressions of *5’-Nase-1* (TEA000333.1) and *5’-Nase-11* (TEA002062.1) are the most abundant in control shoot tips, which are both downregulated after caffeine or theophylline feeding for 48 h in shoot tips compared with the control (feeding for 0 h) (**Figure 7F**). Adenine phosphoribosyltransferase (APRT) catalyzes the conversion of adenine to AMP. Gene expressions of *APRT-1* (TEA007061.1), *APRT-2* (TEA015248.1), *APRT-4* (TEA017916.1), and *APRT-6* (TEA022940.1) decreased significantly after caffeine or theophylline feeding for 48 h in shoot tips compared with the control (feeding for 0 h) (**Figure 7E**).

### Gene Expression Validated by Quantitative Real-Time PCR (QRT-PCR)

To validate the transcriptomic data in our research, relative expressions of 12 selected genes in shoot tips during caffeine or theophylline feeding and degradation were analyzed by qRT-PCR. These genes included nine structural genes involved in caffeine biosynthesis pathway and three genes involved in SAM cycle pathway. Consistent with the transcriptomic datasets, the expression of *TCS*, *MXMT*, *XMT*, *5’-Nase*, *ADK*, *SAMS*, and *SAHH* significantly decreased in shoot tips after fed with caffeine or theophylline for 48 h (**Supplementary Figure S5**). Except *SAMS-4* (TEA020895.1) expression had an increase from theophylline feeding for 48 h to theophylline degradation for 3 days rather than decrease as presented in transcriptomic data, other gene expression changes from caffeine or theophylline feeding for 48 h to caffeine or theophylline degradation for 3 days in tea plant shoot tips were consistent with the transcriptomic data and these genes expressions all decreased during this period. These findings in qRT-PCR could further verify that caffeine or theophylline feeding to tea plant shoot tips significantly inhibited gene expression involved in caffeine biosynthesis pathway and SAM cycle pathway, the latter is crucial to synthesize the methyl donor that is SAM, thus caffeine or theophylline feeding had stimulated a regulation of negative feedback to caffeine biosynthesis in tea plant shoot tips.

### Heterologous Expression of Two MT Genes in E. coli and Enzyme Activity Assay

*TCS-4* (TEA015791.1) and *TCS-8* (TEA028050.1) are the two *MT* genes with the most abundant gene expression in tea plant shoot tips. As elucidated above, the expression levels of *TCS-4* and *TCS-8* decreased significantly in shoot tips fed with caffeine or theophylline, suggesting that these two genes played a crucial role in caffeine biosynthesis. We thus investigated the functions of *TCS-4* and *TCS-8*. The GST (Glutathione *S*-transferase) tagged TCS-4 and TCS-8 were expressed in *E. coli*, using *TCS1* (GenBank: AB031280.1) gene in the same tags expressed in *E. coli* as a positive control.

SDS-PAGE analysis of protein expression in *E. coli* and partial purification were performed, and results showed that GST-TCS-4 and GST-TCS-8 fusion proteins were ~ 68 KDa, including ~ 42 KDa TCS-4 or TCS-8 plus 26 KDa of GST tag, suggesting that these fusion proteins were clearly expressed and partially purified (**Supplementary Figure S6**).

**Figure 8:**
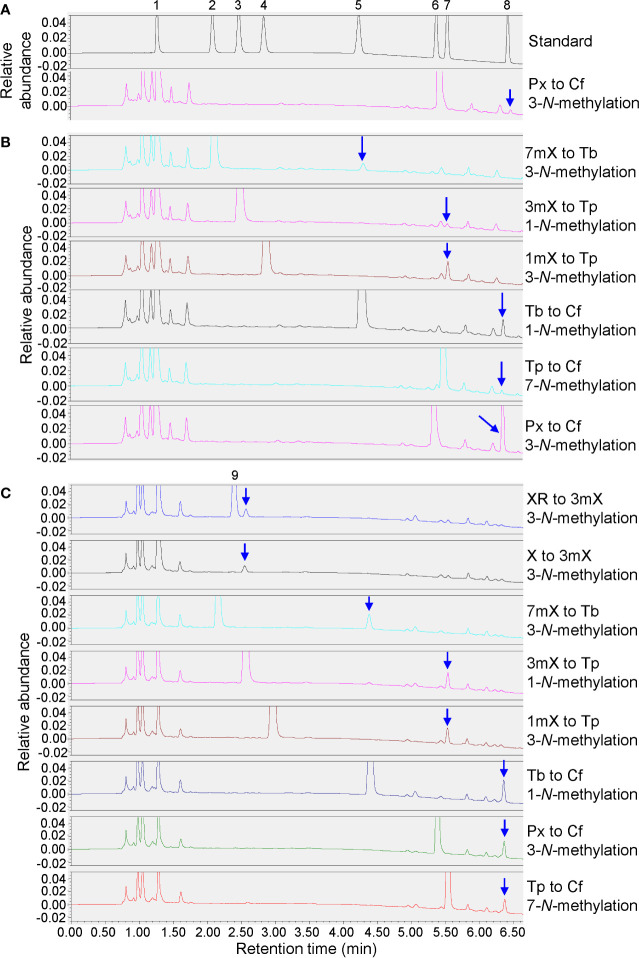
Enzyme activity assays for recombinant genes. **(A)** Enzyme activity assay for recombinant TCS-8-1. 1mM substrate feeding of paraxanthine (Px) to induced cells. 1, xanthine (X); 2, 7-methylxanthine (7mX); 3, 3-methylxanthine (3mX); 4, 1-methylxanthine (1mX); 5, theobromine (Tb); 6, paraxanthine (Px); 7, theophylline (Tp); 8, caffeine (Cf). **(B)** Enzyme activity assay for recombinant TCS-8-2. 1mM substrate feedings of 7-methylxanthine (7mX), 3-methylxanthine (3mX), 1-methylxanthine (1mX), theobromine (Tb), theophylline (Tp), and paraxanthine (Px) to induced cells. **(C)** Enzyme activity assay for recombinant TCS1. 1mM substrate feedings of xanthosine (XR), xanthine (X), 7-methylxanthine (7mX), 3-methylxanthine (3mX), 1-methylxanthine (1mX), theobromine (Tb), paraxanthine (Px), and theophylline (Tp) to induced cells.

Enzyme activity assay showed that the recombinant TCS-8-2 protein exhibited 3-*N*-methyltransferase activity that transformed 7mX (7-methylxanthine) to theobromine, 1mX (1-methylxanthine) to theophylline, or paraxanthine (1,7-dimethylxanthine) to caffeine, as well as 1-*N*-methyltransferase activity that converted 3mX (3-methylxanthine) to theophylline or theobromine to caffeine (**Figure 8B**). Moreover, a weak 7-*N*-methyltransferase activity transforming theophylline to caffeine was also detected (**Figure 8B**). Obviously, TCS-8-2 enzyme displayed most effective activity towards converting paraxanthine to caffeine, followed by the activity converting theobromine to caffeine, and 1mX to theophylline. Except the above enzyme activity, the recombinant TCS1 enzyme could also catalyze the conversions of xanthosine to 3mX, or xanthine to 3mX (**Figure 8C**). However, the recombinant TCS-8-1 enzyme could only catalyze the conversion of paraxanthine to caffeine slowly (**Figure 8A**).

## Discussion

Although caffeine biosynthesis and the key enzymes have been extensively studies and its divergent/convergent evolution have been the hot research spot, caffeine degradation and potential metabolic network remain largely unknown (Ashihara et al., 1997; Huang et al., 2016; Ashihara et al., 2017; Zhu et al., 2019; Zhao et al., 2020). This study integrated purine alkaloid metabolite profiling and transcriptome analysis on caffeine-, theophylline-, and theobromine-fed tea plant shoot tips and roots, and their *in site* degradation during prolonged incubation, further proved that both caffeine biosynthesis and degradation can go through either theobromine or theophylline and 3-MX, under certain circumstances (Ashihara et al., 1997; Huang et al., 2016; Ashihara et al., 2017). The multi-functions of CsTCS-8 enzyme further supported the complex metabolic network and interconversions of the xanthine alkaloids in tea plants.

### Feedback Inhibition on Caffeine Biosynthesis in Both Roots and Shoot Tips Fed With Caffeine and Theophylline

When hydroponically grown tea seedlings or detached tea plant shoot tips were fed with 15 mM caffeine and theophylline, a clear feedback inhibition on caffeine biosynthesis was observed in both roots and shoot tips, particularly for these tea plant tissues fed with caffeine. The feedback inhibition was reflected by immediate decrease in theobromine precursor level in shoot tips after being fed with caffeine for 6 h, and the decreases were lasted till 72 h (**Figures 1B, C**). Theobromine is well-known as a direct precursor for caffeine biosynthesis, through the action of TCSs (Ashihara et al., 1997; Ogita et al., 2004; Jin et al., 2016). The feedback inhibition was also more remarkably reflected by significant repression of the most *TCS* and *MXMT* genes (Ashihara et al., 2006; Mohanpuria et al., 2011). Caffeine feeding of both roots and detached tea plant shoot tips significantly repressed *TCS* and *MXMT* genes (**Figure 7A**). The feeding of 15 mM theophylline to the shoot tips also resulted in clear decrease in theobromine contents. Interestingly, 15 mM theophylline to the shoot tips also increased caffeine contents, perhaps due to the repression of caffeine catabolism into theophylline. However, it could be due to parts of theophylline were converted into caffeine through 3mX and theobromine, as indicated by previous tracer experiments (Ashihara et al., 1997). In support of the observation, we observed that theophylline feeding of the shoot tips resulted much less repression of caffeine biosynthetic genes compared with caffeine feeding to shoot tips, such as *TCS-4* (TEA015791.1), *TCS-8* (TEA028050.1), *MXMT-5* (TEA028051.1), and *MXMT-3* (TEA012581.1). Evidence is also shown that theophylline feeding resulted in faster degradation into 3mX, which was accumulated to the higher levels in theophylline-fed shoot tips than caffeine-fed ones (**Figures 1C** and **4B**). Therefore, although theobromine levels were decreased in theophylline-fed shoot tips, more caffeine were synthesized by TCSs.

### Interconversion Between Caffeine, Theobromine, Theophylline, and 3mX in Tea Plants

Previous studies have clearly showed that a major routine for caffeine degradation in tea plants are *via* caffeine, theophylline, 3mX, X, and other intermediates, uric acid, allantoin, allantoic acid, ureidoglycolic acid, urea to NH_3_, CO_2_, and glyoxylic acid (Ashihara et al., 1997; Muñoz et al., 2006; Ashihara et al., 2017). Our feeding experiments and metabolite profiling on tea plant roots showed that caffeine absorbed into the roots can be demethylated into theobromine, which is consistent with previous report (Zhu et al., 2019). Feeding of roots or shoot tips with 15 mM theophylline also indicated that it could be converted into caffeine and 3mX in shoot tips, and degraded into 3mX and then converted into theobromine, and then into caffeine in roots. Although previous studies have shown that theophylline in young shoot tips can be traced into caffeine through 3mX by a 1-*N* demethylase and then 7-*N* methylation into theobromine by a 7-*N*-methyl transferase. It could also not be excluded that a possible reversible conversion between theophylline and caffeine by 7-*N* methylation. Also, feeding experiments with theobromine of roots also showed that theobromine could be either 3-*N* demethylated into 7mX and 7-*N* demethylation into X, or 1-*N* methylation by TCSs into caffeine. Thus, tea plant tissues own capability to inter-convert caffeine, theobromine, theophylline, and 3mX through methylation and de-methylation reactions, although the enzymes catalyzing parts of these methylation reactions are known, most of de-methylation reactions remain unknown.

### Tea Plant Roots Could Degrade Caffeine More Efficiently Than Shoot Tips Did

After fed with 15 mM caffeine or theophylline or 3 mM theobromine for up to 72 h, all these hydroponically grown tea plant seedling roots and detached shoot tips had taken up and accumulated significant levels of these purine alkaloids. The degradation experiments on these purine alkaloids in roots or shoot tips for up to 5 days indicated that, caffeine was indeed very stable in shoot tips, only 10.4% of total caffeine were degraded; whereas 44.5% of total absorbed caffeine in roots were degraded in roots, strongly suggesting that tea plant roots had more efficient caffeine degradation enzymes than shoot tips did. However, for theophylline degradation, the degradation rate of absorbed theophylline in roots was 51.7%, slightly higher than that of shoot tips, 46.8%. These results are consistent with previous observations, that theophylline is more efficiently degraded than caffeine is, and caffeine degradation into theophylline could be the rate-limiting step in caffeine degradation (Ashihara et al., 1996; Ashihara et al., 1997; Ashihara and Crozier, 2001; Ashihara et al., 2017). The previous using tracer experiments showed that caffeine almost could not be degraded with any detectable degradation product (Ashihara et al., 1996; Ashihara et al., 1997). However, 75% theophylline could be degraded into 3mX, and X in mature and old tea plant leaves, whereas majority of fed theophylline in young leaves was incorporated into 3mX, theobromine, and caffeine (Ashihara et al., 1997). We also observed a faster appearance of 3mX in roots than in shoot tips when fed with theophylline.

Tea pant roots usually accumulate very low levels of caffeine or other purine alkaloids, and this could be not only due to the significantly lower biosynthesis rate of caffeine, as reflected by lower expression of critical *TCS* and *MXMT* genes in roots, but also due to the much stronger caffeine degradation capability in roots than in shoot tips. Thus, when fed with 15 mM caffeine and theophylline, roots showed more sensitive responses to high concentration of purine alkaloids, and more differentially expressed genes (DEGs) (**Supplementary Figure S4**). DEG numbers in different comparisons suggested that tea plant roots had more sensitive and stronger response to the toxic levels of these purine alkaloids. Such stronger physiological responses and extensive gene expression changes could provide bases for the more efficient caffeine and theophylline degradation in roots rather than in shoot tips.

While shoot tips had higher capacity to synthesize and accumulate more caffeine, theobromine, fed with 15 mM caffeine or theophylline caused much significant feedback inhibition effects than roots did. This was mainly reflected by more significantly repressed TCS and MXMT gene expression. Furthermore, such repression of *TCS* and *MXMT* genes lasted for much longer in shoot tips. Except for *MXMT-7* (TEA030024.1), expression levels of most *TCS* and *MXMT* genes continuously decreased from the beginning of feeding to 48 h after feeding of caffeine and theophylline, to degradation for 3 days.

For examples, two *TCS* genes, *TCS-4* (TEA015791.1) and *TCS-8* (TEA028050.1) were the most highly expressed ones in shoot tips, apical buds, and young leaves. Compared with un-fed shoot tips, these two genes in caffeine-fed shoot tips were repressed by about 3.8- and 3-fold, respectively, at 48 h after feeding. *TCS-4* and *TCS-8* expression levels in theophylline-fed shoot tips were reduced by 3.2- and 2.6-fold, respectively, at 48 h after feeding. On the contrary, compared with that in non-fed control roots, *TCS-4* expression in caffeine-fed roots even increased by 2.5-fold at 12 h after feeding, and then decreased by 2-fold at 48 h after feeding. *TCS-8* was also repressed slightly in roots fed with caffeine or theophylline.

### Caffeine Degradation in Tea Plant Shoot Tips and Roots

As caffeine is relatively stable in tea plant shoot tips, many green teas contain high levels of caffeine, and cause sleeping problems to tea drinkers. Consistent with previous studies (Ashihara et al., 1996; Ashihara et al., 1997), caffeine in shoot tips was also barely degraded in our feeding and degradation study (**Figures 1A, C**). However, a significant portion of caffeine was degraded in roots after absorption and accumulation of high levels of caffeine. Thus, it could still be a good system to study caffeine degradation by tea plants.

**Figure 9:**
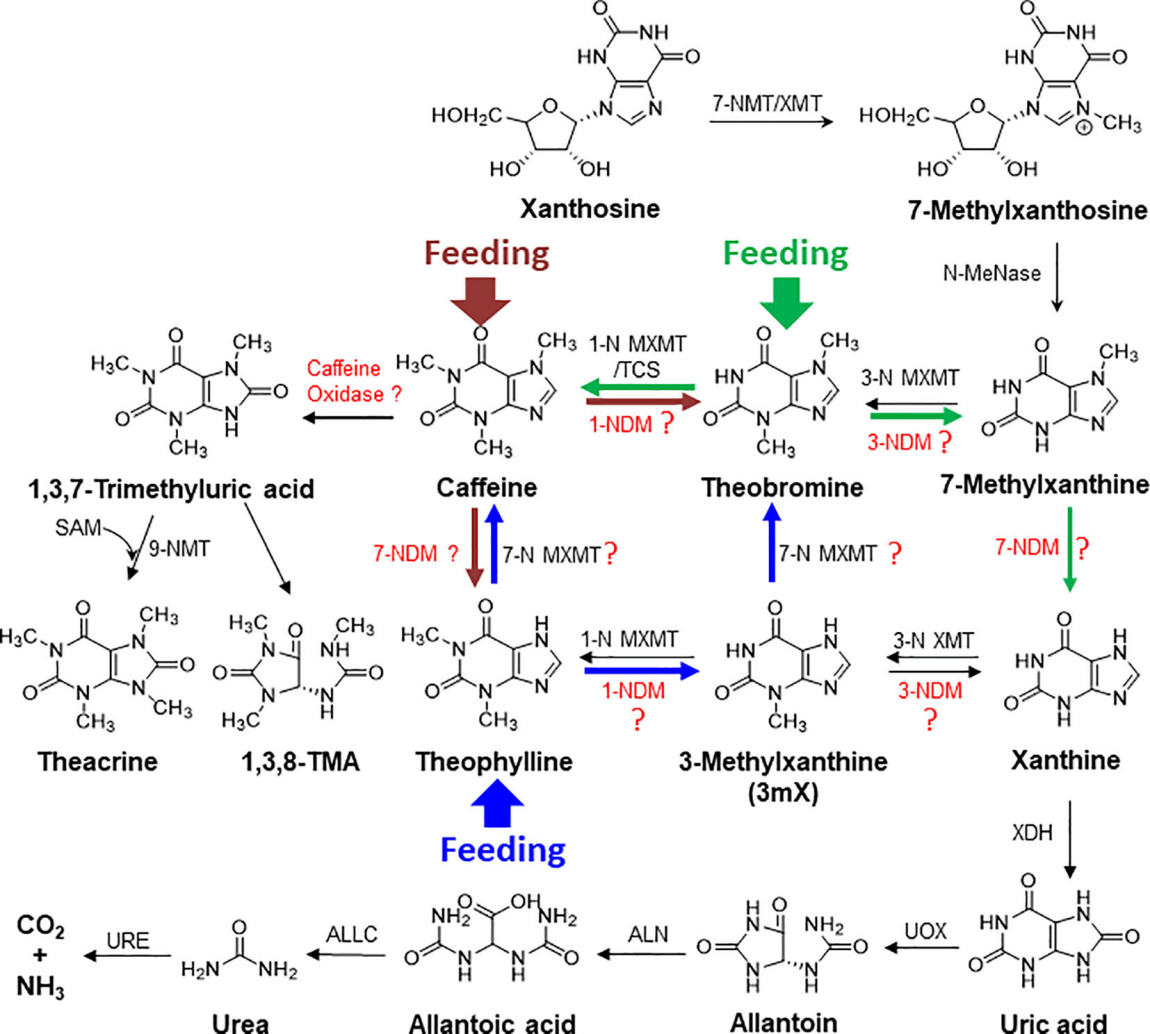
The proposed complex metabolic network for purine alkaloid biosynthesis and degradation in tea plant. The brown, blue, and green arrows represent the detected metabolic flux in feeding of caffeine, theophylline, and theobromine, respectively, to tea plant roots and shoot tips. XMT, xanthione methyltransferase; MXMT, methylxanthine methyltransferase; NDM, N-demethylase; XDH, xanthine dehydrogenase; UOX, urate oxidase; ALN, allantoinase; URE, urease; ALLC, allantoicase.

Caffeine acts as antimicrobial and neurotoxin to kill microbes and to repel herbivores, and thus is used to engineer disease- and insect-resistant crops (Hollingsworth et al., 2002; Uefuji et al., 2005; Kim and Sano, 2008). Plants, such as tea, cacao, or coffee, may evolve caffeine as an anti-insect and anti-microbial chemical to build their innate immunity. In co-evolution, many microbes had also evolved efficient caffeine degradation enzymes (Mazzafera, 2004; Mohapatra et al., 2006; Yu et al., 2008; Yu et al., 2009; Ashihara et al., 2017). Thus, so far enzymes for catabolism of caffeine are primarily discovered in microorganisms *via* two mechanisms: demethylation and oxidation: the major demethylation metabolite formed in fungi is theophylline whereas theobromine is the major demethylation metabolite in bacteria (Dash and Gummadi, 2006). The proposed enzymes, such as 1-*N*, 3-*N*, and 7-*N*-demethylase P450s, are not characterized from tea, cacao, or coffee plants, and theophylline can be relative efficiently converted into xanthine, by demethylases or other degradation enzymes (**Figure 9**) (Ashihara et al., 2017; Zhao et al., 2020). BLAST search against tea plant genome only obtained some P540 genes with very low similarity. Caffeine can be oxidized into trimethyl uric acid in a single step in some bacteria (Dash and Gummadi, 2006). Interestingly, theacrine found in kucha may be as a derivative of caffeine through oxidization at 8-*C*, resulting in 1,3,7-trimethyluric acid, which is further methylated at 9-*N* (**Figure 9**) (Zhang et al., 2020). Recently, 1,3,8-trimethylallantoin was found in tea plants, even in low amounts (Wang et al., 2019), suggesting that caffeine oxidase-catalyzed 1,3,7-trimethyluric acid could be a natural caffeine degradation pathway (**Figure 9**). Understanding of caffeine degradation mechanism may facilitate the breeding of low-caffeine or -theobromine tea plant varieties for meeting diverse demands. The study lays foundations for our further exploration of caffeine degradation in tea plants.

## Data Availability Statement

The original contributions presented in the study are publicly available. This data can be found here in NCBI BioProject: PRJNA649944 https://dataview.ncbi.nlm.nih.gov/object/PRJNA649944?reviewer=o39fqtahfk5sc8du04dd5bpe34.

## Author Contributions

ZZ, JZ, and WD guided the research. CD, XK, LC, S-AP, and LF performed the research. CD analyzed the data. JZ and CD wrote the manuscript. All authors contributed to the article and approved the submitted version.

## Funding

This work was supported by the National Natural Science Foundation of China (31570692), the National Key Research and Development Program of China (2018YFD1000601), the Changjiang Scholars and Innovative Research Team in University (IRT_15R01), and the Key Research and Development (R&D) Program of Anhui Province (18030701155).

## Conflict of Interest

The authors declare that the research was conducted in the absence of any commercial or financial relationships that could be construed as a potential conflict of interest.

## Supplementary Material

Supplementary Table S1 Primers used in this study.

Supplementary Table S2 Gene expression of identified caffeine biosynthesis genes in eight tissues [apical vegetative bud (AB); young, mature and old leaves (YL, ML, OL); root (RT); stem (ST); flower (FL) and fruit (FR)].

Supplementary Table S3 GenBank accession numbers of genes in the phylogenetic tree.

Supplementary Table S4 Differential expression levels (FPKM values) of genes involved in caffeine biosynthesis and catabolic pathway in caffeine- and theophylline-fed shoot tips and roots.
