# Supplementary material for: Metabolite and Transcriptome Profiling on Xanthine Alkaloids-Fed Tea Plant (Camellia sinensis) Shoot Tips and Roots Reveal the Complex Metabolic Network for Caffeine Biosynthesis and Degradation
Source: Front Plant Sci. 2020 Sep 9;11:551288. doi: 10.3389/fpls.2020.551288 (PMC7509060; doi:10.3389/fpls.2020.551288)
Supplement: Supplementary file 1 [file DataSheet_1.pdf]

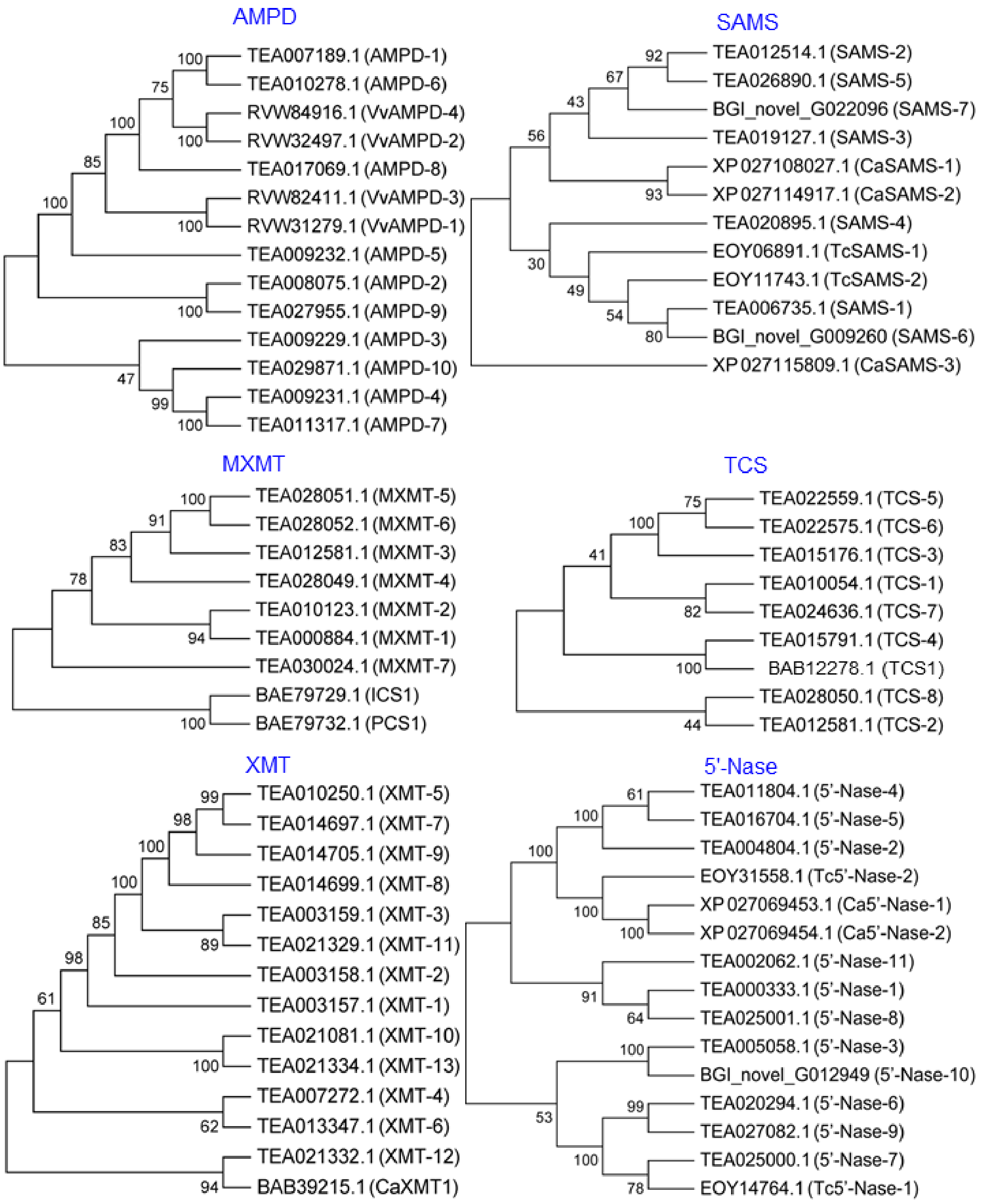
AMPD
TEA007189.1 (AMPD-1)
TEA010278.1 (AMPD-6)
RVW84916.1 (VvAMPD-4)
RVW32497.1 (VvAMPD-2)
TEA017069.1 (AMPD-8)
RVW82411.1 (VvAMPD-3)
RVW31279.1 (VvAMPD-1)
TEA009232.1 (AMPD-5)
TEA008075.1 (AMPD-2)
TEA027955.1 (AMPD-9)
TEA009229.1 (AMPD-3)
TEA029871.1 (AMPD-10)
TEA009231.1 (AMPD-4)
TEA011317.1 (AMPD-7)
SAMS
TEA012514.1 (SAMS-2)
TEA026890.1 (SAMS-5)
BGI_novel_G022096 (SAMS-7)
TEA019127.1 (SAMS-3)
XP 027108027.1 (CaSAMS-1)
XP 027114917.1 (CaSAMS-2)
TEA020895.1 (SAMS-4)
EOY06891.1 (TcSAMS-1)
EOY11743.1 (TcSAMS-2)
TEA006735.1 (SAMS-1)
BGI_novel_G009260 (SAMS-6)
XP 027115809.1 (CaSAMS-3)
MXMT
TEA028051.1 (MXMT-5)
TEA028052.1 (MXMT-6)
TEA012581.1 (MXMT-3)
TEA028049.1 (MXMT-4)
TEA010123.1 (MXMT-2)
TEA000884.1 (MXMT-1)
TEA030024.1 (MXMT-7)
BAE79729.1 (ICS1)
BAE79732.1 (PCS1)
TCS
TEA022559.1 (TCS-5)
TEA022575.1 (TCS-6)
TEA015176.1 (TCS-3)
TEA010054.1 (TCS-1)
TEA024636.1 (TCS-7)
TEA015791.1 (TCS-4)
BAB12278.1 (TCS1)
TEA028050.1 (TCS-8)
TEA012581.1 (TCS-2)
XMT
TEA010250.1 (XMT-5)
TEA014697.1 (XMT-7)
TEA014705.1 (XMT-9)
TEA014699.1 (XMT-8)
TEA003159.1 (XMT-3)
TEA021329.1 (XMT-11)
TEA003158.1 (XMT-2)
TEA003157.1 (XMT-1)
TEA021081.1 (XMT-10)
TEA021334.1 (XMT-13)
TEA007272.1 (XMT-4)
TEA013347.1 (XMT-6)
TEA021332.1 (XMT-12)
BAB39215.1 (CaXMT1)
5'-Nase
TEA011804.1 (5'-Nase-4)
TEA016704.1 (5'-Nase-5)
TEA004804.1 (5'-Nase-2)
EOY31558.1 (Tc5'-Nase-2)
XP 027069453.1 (Ca5'-Nase-1)
XP 027069454.1 (Ca5'-Nase-2)
TEA002062.1 (5'-Nase-11)
TEA000333.1 (5'-Nase-1)
TEA025001.1 (5'-Nase-8)
TEA005058.1 (5'-Nase-3)
BGI_novel_G012949 (5'-Nase-10)
TEA020294.1 (5'-Nase-6)
TEA027082.1 (5'-Nase-9)
TEA025000.1 (5'-Nase-7)
EOY14764.1 (Tc5'-Nase-1)

Figure S1

Figure S1. Phylogenetic trees of the genes involved in caffeine biosynthetic pathway. Maximum likelihood (ML) tree for each caffeine biosynthetic gene was constructed using MEGA 7.0 with 500 bootstrap replicates. CDS protein sequences of AMPD (AMP deaminase), SAMS (*S*-adenosyl-L-methionine synthase), MXMT (7-methylxanthine methyltransferase), TCS (tea caffeine synthase), XMT (xanthosine methyltransferase) and 5'-Nase (5'-nucleotidase) are from *Camellia sinensis*, the full names of abbreviations ahead of the protein names are as follows: Vv (*Vitis vinifera*), Ca (*Coffea arabica*), Tc (*Theobroma cacao*). ICS: theobromine synthase in *Camellia irrawadiensis*; PCS: theobromine synthase in *Camellia ptilophylla.*

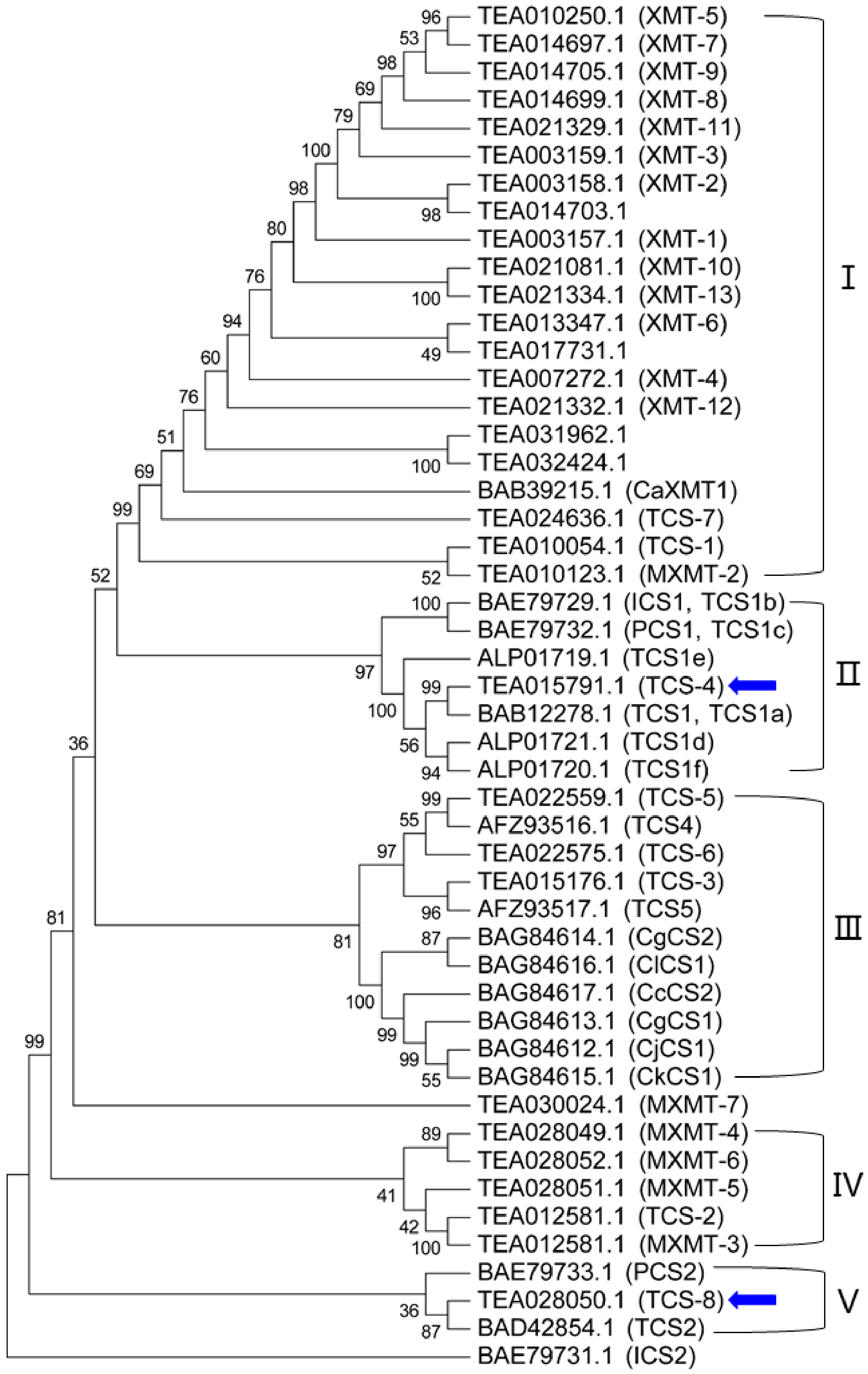
TEA010250.1 (XMT-5)
TEA014697.1 (XMT-7)
TEA014705.1 (XMT-9)
TEA014699.1 (XMT-8)
TEA021329.1 (XMT-11)
TEA003159.1 (XMT-3)
TEA003158.1 (XMT-2)
TEA014703.1
TEA003157.1 (XMT-1)
TEA021081.1 (XMT-10)
TEA021334.1 (XMT-13)
TEA013347.1 (XMT-6)
TEA017731.1
TEA007272.1 (XMT-4)
TEA021332.1 (XMT-12)
TEA031962.1
TEA032424.1
BAB39215.1 (CaXMT1)
TEA024636.1 (TCS-7)
TEA010054.1 (TCS-1)
TEA010123.1 (MXMT-2)
BAE79729.1 (ICS1, TCS1b)
BAE79732.1 (PCS1, TCS1c)
ALP01719.1 (TCS1e)
TEA015791.1 (TCS-4)
BAB12278.1 (TCS1, TCS1a)
ALP01721.1 (TCS1d)
ALP01720.1 (TCS1f)
TEA022559.1 (TCS-5)
AFZ93516.1 (TCS4)
TEA022575.1 (TCS-6)
TEA015176.1 (TCS-3)
AFZ93517.1 (TCS5)
BAG84614.1 (CgCS2)
BAG84616.1 (ClCS1)
BAG84617.1 (CcCS2)
BAG84613.1 (CgCS1)
BAG84612.1 (CjCS1)
BAG84615.1 (CkCS1)
TEA030024.1 (MXMT-7)
TEA028049.1 (MXMT-4)
TEA028052.1 (MXMT-6)
TEA028051.1 (MXMT-5)
TEA012581.1 (TCS-2)
TEA012581.1 (MXMT-3)
BAE79733.1 (PCS2)
TEA028050.1 (TCS-8)
BAD42854.1 (TCS2)
BAE79731.1 (ICS2)
I
II
III
IV
V

Figure S2

Figure S2. Phylogenetic tree of Methyltransferases (MTs) CDS protein sequences from *Camellia* and *Coffea* plants. XMT: xanthosine methyltransferase; CaXMT1: xanthosine methyltransferase in *Coffea arabica*; TCS: tea caffeine synthase; MXMT: 7-methylxanthine methyltransferase; ICS: theobromine synthase in *Camellia irrawadiensis*; PCS: theobromine synthase in *Camellia ptilophylla*; *TCS1a-f* are six types of allelic variations of *TCS1*. TCS1a-f, TCS2-5, ICS2, and PCS2 are all from section *Thea*, CjCS1 from *Camellia. japonica*, CgCS1 and CgCS2 from *C. granthamiana*, ClCS1 from *C. lutchuensis*, CkCS1 from *C. kissi*, and CcCS2 from *C. chrysantha*. The phylogenetic tree was constructed using MEGA 7.0 with the neighbor-joining method. TCS-4 and TCS-8, downregulated significantly after fed with caffeine or theophylline, marked with a blue arrow.

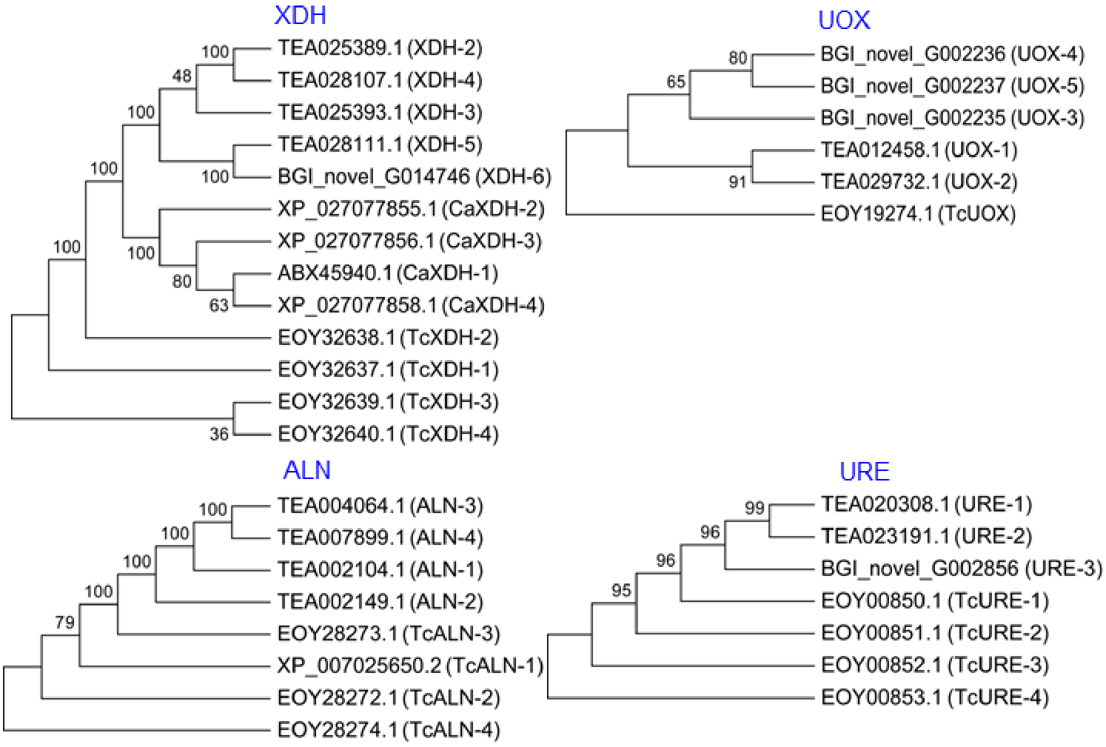

Figure S3

Figure S3. Phylogenetic trees of the genes involved in caffeine catabolic pathway. Maximum likelihood (ML) tree for each caffeine biosynthetic gene was constructed using MEGA 7.0 with 500 bootstrap replicates. CDS protein sequences of XDH (xanthine dehydrogenase), UOX (urate oxidase), ALN (allantoinase) and URE (urease) are from *Camellia sinensis*, the full names of abbreviations ahead of the protein names are as follows: Ca (*Coffea arabica*), Tc (*Theobroma cacao*).

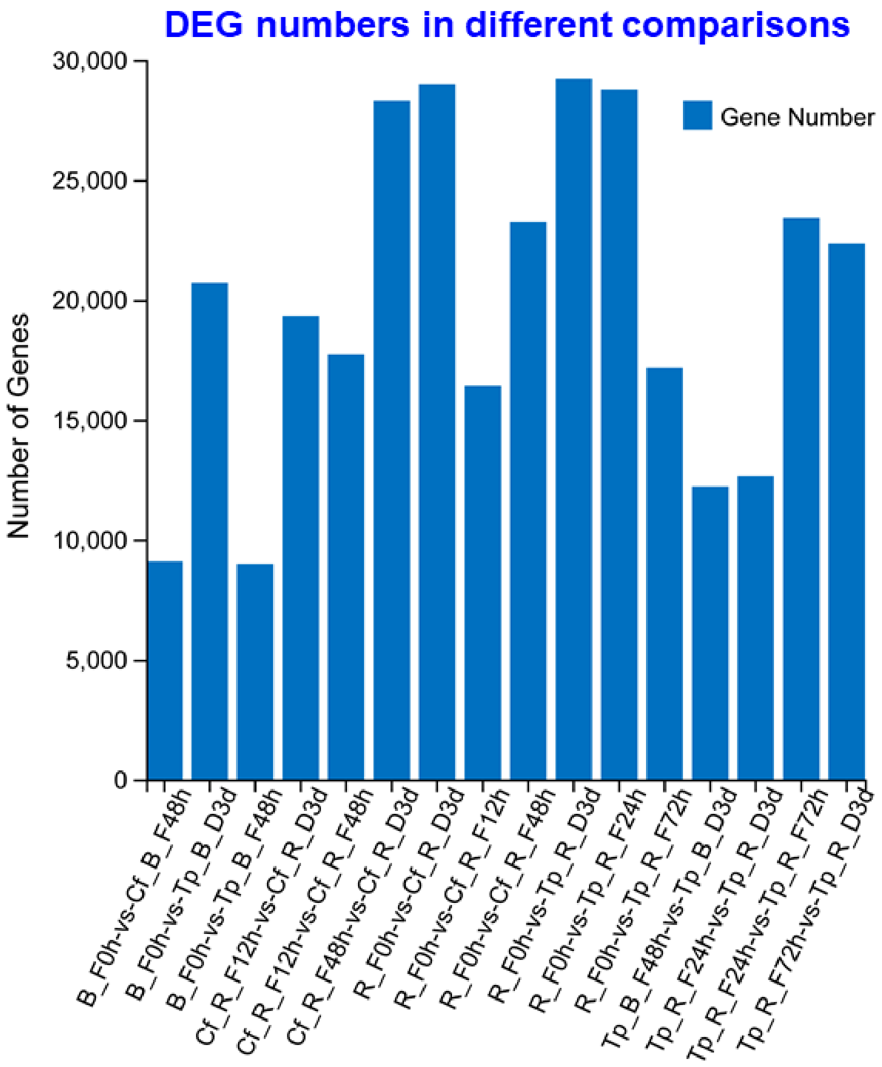

Figure S4

Figure S4. DEG numbers in different comparisons. The X-axis represents the difference comparison scheme of each group, and the Y-axis represents the corresponding DEG numbers.

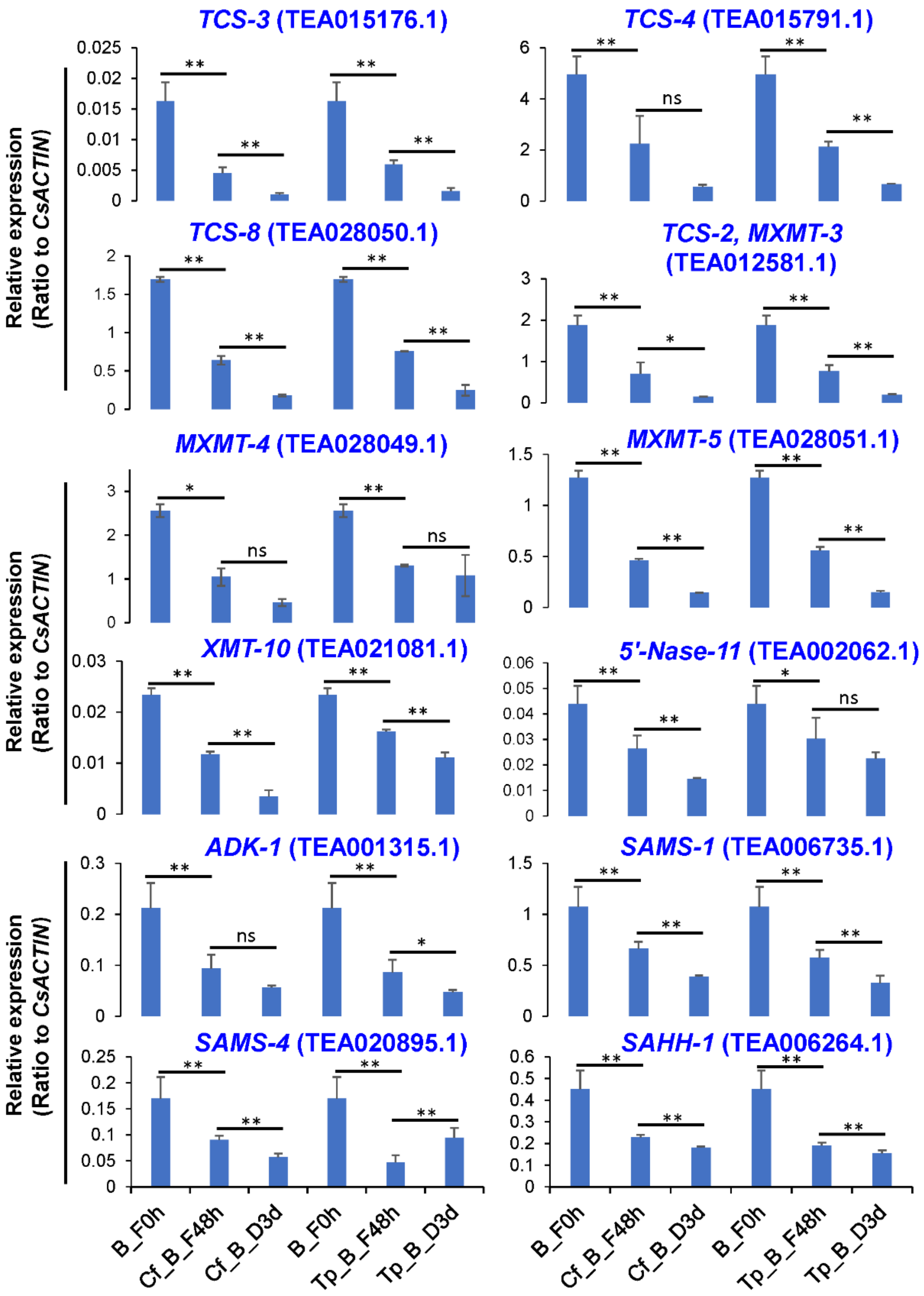

TCS-3 (TEA015176.1)
TCS-4 (TEA015791.1)
TCS-8 (TEA028050.1)
TCS-2, MXMT-3 (TEA012581.1)
MXMT-4 (TEA028049.1)
MXMT-5 (TEA028051.1)
XMT-10 (TEA021081.1)
5'-Nase-11 (TEA002062.1)
ADK-1 (TEA001315.1)
SAMS-1 (TEA006735.1)
SAMS-4 (TEA020895.1)
SAHH-1 (TEA006264.1)
Relative expression (Ratio to CsACTIN)
B_F0h
Cf_B_F48h
Cf_B_D3d
B_F0h
Tp_B_F48h
Tp_B_D3d

Figure S5

Figure S5. Validation of gene expression in transcriptomic data regarding of caffeine synthesis. Data are presented as means ± SD from at least three independent repeats. Two-tailed Student's *t*-tests were performed to compare data at each time point against the control (0 h or 48 h). Significant differences are labeled with asterisks (*P < 0.05, **P < 0.01), ns represents for no significance.

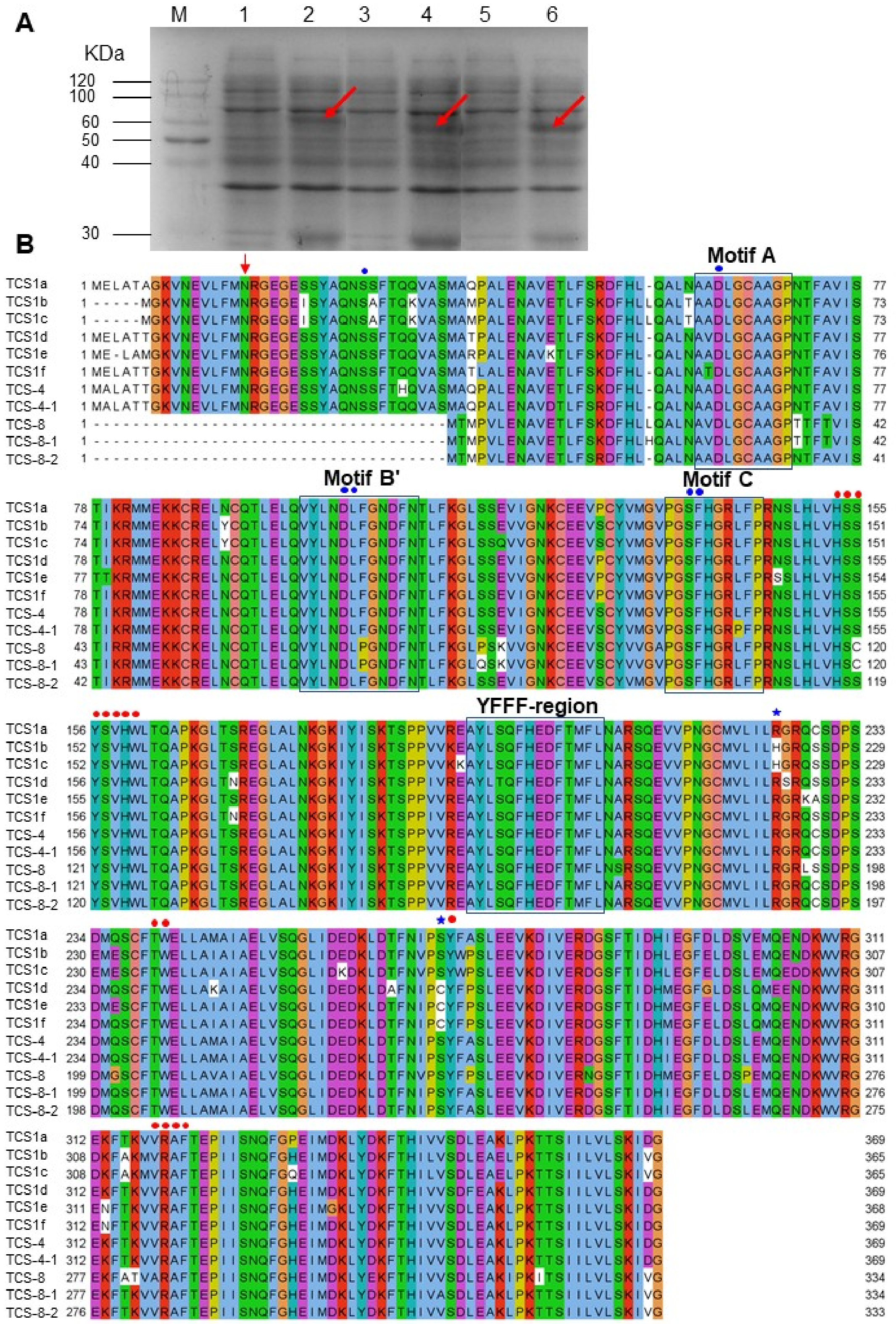
A
M
1
2
3
4
5
6
KDa
120
100
60
50
40
30
B
Motif A
Motif B'
Motif C
YFFF-region
TCS1a
TCS1b
TCS1c
TCS1d
TCS1e
TCS1f
TCS-4
TCS-4-1
TCS-8
TCS-8-1
TCS-8-2

Figure S6

Figure S6. (A) SDS-PAGE analysis of protein extracts from *E. coli* expressing TCS-4-GST and TCS-8-GST fusion proteins. Lane M, standard protein ladder. Lane 1 and 2, crude protein from uninduced cells and induced cells of TCS-4-1. Lane 3 and 4, crude protein from uninduced cells and induced cells of TCS-8-1. Lane 5 and 6, crude protein from uninduced cells and induced cells of TCS-8-2. Red arrows indicate TCS-4-GST and TCS-8-GST fusion proteins.

(B) Multiple sequences alignment of tea caffeine synthase (*TCS*) genes. *TCS1a-f* are six types of allelic variations of *TCS1*. *TCS-4* and *TCS-8* are TEA015791.1 and TEA028050.1 respectively. The proposed SAM-binding motifs (A, B', and C) and conserved "YFFF-region" are shown by rectangular boxes. Substrate binding sites of methyl acceptor and methyl donor (SAM) are marked by ● and ● respectively. Additional active site residue is marked by red arrowhead. The amino acid residues marked by blue pentagram play a critical role in substrate recognition.
